# SGK1 aggravates idiopathic pulmonary fibrosis by triggering H3k27ac-mediated macrophage reprogramming and disturbing immune homeostasis

**DOI:** 10.7150/ijbs.90808

**Published:** 2024-01-12

**Authors:** Jianzhi Wu, Liping Gong, Yijie Li, Tiegang Liu, Rong Sun, Kexin Jia, Runping Liu, Fei Dong, Xiaohong Gu, Xiaojiaoyang Li

**Affiliations:** 1 School of Life Sciences, Beijing University of Chinese Medicine, Beijing, 100029, China.; 2 Institute of Chinese Epidemic Disease, Beijing University of Chinese Medicine, Beijing 100029, China.; 3 The Second Hospital of Shandong University, Shan Dong University, 247 Bei Yuan Da Jie, Jinan, 250033, China.; 4 School of Chinese Materia Medica, Beijing University of Chinese Medicine, 11 Bei San Huan Dong Lu, Beijing, 100029, China.; 5 School of Traditional Chinese Medicine, Beijing University of Chinese Medicine, Beijing, 100029, China.

**Keywords:** SGK1, pulmonary fibrosis, macrophage reprogramming, H3K27ac, immune homeostasis

## Abstract

Idiopathic pulmonary fibrosis (IPF) is characterized by fibrotic matrix deposition and irreversible aberrant tissue remodeling. Their mechanisms of action are associated with the activation of macrophages and a disturbed immune environment. We aim to determine how these activated macrophages influenced the pathogenesis of pulmonary fibrosis. We found the fibrotic areas of IPF patients contained more serum and glucocorticoid-induced kinase 1 (SGK1)-positive and M2-type macrophages. Similarly, bleomycin (BLM)+LPS significantly triggered high expression of SGK1 in the IPF mice, accompanied by destroyed lung structure and function, increased fibrosis markers and disturbed immune microenvironment. Mechanistically, SGK1 markedly promoted the reprogramming of M2-type macrophages in fibrotic lungs by triggering glycogen synthase kinase 3beta (GSK3β)-tat-interacting protein 60 (TIP60)- histone-3 lysine-27 acetylation (H3K27ac) signalings, which further released chemokine (C-C motif) ligand 9 (CCL9) to attract Th17 cells and delivered TGF-β to fibroblasts for synergistically destroying immune microenvironment, which was largely reversed by macrophage depletion in mice. We took macrophages as the entry point to deeply analyze IPF pathogenesis and further provided insights for the development of novel drugs represented by SGK1.

## 1. Introduction

Idiopathic pulmonary fibrosis (IPF) is a progressive and irreversible lung disease characterized by the secretion of proinflammatory mediators, the destruction of alveolar architecture, the aberrant repair of the injured alveolus and following overwhelming deposition of interstitial fibro 1. Thus far, a variety of insults like toxic drugs, autoimmune disorders or traumatic injuries, are significant etiological factors of IPF. Due to limited effective therapies and unclear pathological mechanisms, IPF may further deteriorate into lung cancer, representing an important source of morbidity and mortality worldwide and imposing a substantial cost burden on patients 2. In addition, several classic animal models respectively induced by bleomycin (BLM), silica or asbestos, has improved our understanding of the pathogenesis of IPF and facilitated the development of potential therapies, such as pirfenidone and nintedanib that obtained good preclinical results 3. However, these Food and Drug Administration (FDA)-approved drugs can only modestly relief the respiratory syndromes but fail to reverse fibrosis progression 4. Recently, other investigators and we have been committed to building novel integrated IPF mouse models such as intervening with lipopolysaccharide (LPS) in the early stage of BLM-induced pulmonary fibrosis to better recapitulate the clinicopathologic features of IPF 5. These improved IPF models will provide better platform for further studies on the specific molecular mechanisms of pathogenesis.

The uncontrolled immune responses for driving the initiation and progression of IPF are receiving increasing attention 6, 7. As the most abundant immune cells (approximately 70%) in the lung, macrophages have been considered to be relevant to IPF pathogenesis for their highly plastic characteristic that can transform toward different active states including classically pro-inflammatory phenotype (M1) and alternatively pro-fibrotic phenotype (M2) 8. Generally, excessive M2 macrophages-associated responses are crucial drivers of continuous activation of fibroblasts that differentiate into myofibroblasts *via* secreting profibrotic mediators such as transforming growth factor-beta (TGF-β), interleukin-1 beta (IL-1β) and platelet derived growth factor (PDGF) 9, 10. Of note, multiple chemokines from activated macrophages also influence the recruitment and the function of other immune T cells, including multiple subsets like T helper cells, regulatory T cells, memory T cells and other innate T-cell subsets, which are also indispensable for the progression of IPF 11. Interestingly, there is another compelling evidence that IL-17A released by T helper 17 (Th17) cells can induce the activation of M2-type macrophages, which may create a perpetuating vicious cycle 12. These above results encouraged us to explore the role of activated macrophages and their interactions with other immune T cells during IPF.

Serum and glucocorticoid-induced kinase 1 (SGK1) belongs to the protein kinase "AGC" subfamily that participates in the pathophysiology of a variety of diseases including multiple fibrosis diseases, lung cancer and other types of malignancies 13-15, thus making SGK1 to be a potential prognostic indicator and therapeutic target. Recently, researchers gradually realized that SGK1 might also be involved in a variety of inflammatory diseases and the upregulated SGK1 was reported to take place primarily in alveolar macrophages and to induce macrophage infiltration and inflammation in the lung by robustly increasing the expressions of crucial cytokines, such as *Il6*, tumour necrosis factor alpha (*Tnfa*), and *Il1b* 16, 17. In addition, the degradation of SGK1 resulted in protective effects against pulmonary fibrotic events 18, 19. Considering the crucial roles of SGK1, the upstream and downstream regulatory pathways that affect SGK1 are also crucial for regulating physiological functions and pathological processes. For instance, SGK1 suppresses the activity of its downstream targets such as glycogen synthase kinase 3beta (GSK3β) and further controls numerous gene expressions 20. Although there is insufficient evidence to support that SGK1 may also be involved in epigenetic regulation process, the regulation of pulmonary fibrosis-related gene expression at the transcriptional level through chromatin and histone modification has attracted more attention 21. Meanwhile, SGK1 also can be regulated by GSK3β and its downstream signaling pathways, forming a complex network 22. Collectively, whether and how SGK1 affects the phenotype of the macrophages in the injured immune microenvironment to promote IPF progression remains obscure.

In the current study, we demonstrated that SGK1 was drastically elevated both in the lungs of pulmonary fibrosis patients and IPF mouse model, and was strongly correlated with disease severity. Mechanically, BLM promoted the acetylation of histone acetylation of histone-3 lysine-27 (H3K27) and subsequent the reprogramming of macrophages from M1 to M2 state by directly activating SGK1 and GSK3β- tat-interacting protein 60 (TIP60) pathways in an LPS-assisted manner. Meanwhile, M2-type macrophages could release chemokine (C-C motif) ligand 9 (CCL9) to recruit immune Th17 cells as well as secret TGF-β to stimulate fibroblast activation, thereby collaboratively exacerbating IPF progression. Our research is expected to provide a better understanding of the molecular mechanisms of IPF as well as promote the development of therapeutic drugs.

## 2. Materials and methods

### 2.1 Materials

BLM sulfate (S17100) was obtained from Shanghai Yuanye Bio-Technology Co., Ltd. LPS (BN32880) was purchased from Biorigin (Beijing) Inc. Phorbol 12-myristate 13-acetate (PMA), EMD638683 (EMD), MG149 were purchased from MCE (Shanghai, China). Dexamethasone (Dex) (D137736) was purchased from Aladdin Ltd, Shanghai, China. All cell culture supplemental components were purchased from Sigma-Aldrich (St. Louis, USA). The antibodies used in this study were listed in the **Table S2**.

### 2.2 Human samples

Clinical studies were approved by the Research Ethics Committee of the Second Hospital of Shandong University (Approval Number: KYLL-2023LW003) and the need for patient approval and/or informed consent was waived due to the retrospective nature of the study. The clinical research followed the ethical principles of the Declaration of Helsinki, the International Conference on Harmonization Guidelines for Good Clinical Practice. IPF was diagnosed according to the American Thoracic Society/European Respiratory Society consensus criteria 23. Lung tissue samples (n = 27) from the remaining tissues after pathological examination were collected by lung surgery in the Second Hospital of Shandong University between January 2021 and October 2022. Control (non-IPF) lung samples were obtained from normal lung tissues surrounding fibrotic tissues. In this research, we evaluated all clinical samples and randomly selected samples of normal or patients with pulmonary fibrosis for further study. The baseline characteristics of the IPF patients are provided in **Table S1**.

### 2.3 Animal study

All procedures for animal studies were approved by the Institutional Animal Care and Use Committee of Beijing University of Chinese Medicine (BUCM-4-2022031005-1094). C57BL/6J mice (8-week-old male, 22-24 g) were purchased from Vital River Laboratory Animal Technology. Mice were housed in a consistent environment (12-hour light/dark cycle, 22 ± 2 °C) with a humidity of 40 ± 10 % and a standard food and sterile water ad *libitum*. To establish chronic lung fibrosis model, mice were randomly separated into 4 groups (n = 6): (1) Control (Con) group; (2) BLM group; (3) LPS group; (4) BLM + LPS group. In brief, BLM-treated mice were treated with BLM (2.5 mg/kg, dissolved in PBS) at day 1 and LPS-treated mice were treated with LPS (1 mg/kg, dissolved in PBS) at days 5, 7 and 9* via* intratracheal. BLM + LPS treated mice were administered with BLM intratracheally at day 1 and then continually received LPS treatment intratracheally at days 5, 7 and 9, as previously described 5. In addition, mice in the Con group were given with PBS intratracheally at each time point. At day 21, mice were anesthetized to test pulmonary functions indicators and then, the serum of mice were collected and sacrificed to harvest lung samples for further experiments. For macrophage deletion experiment, C57BL/6J mice were randomly separated into 4 groups (n = 6): (1) con empty liposome group; (2) clodronate liposomes (Clo) group; (3) BLM + LPS + control empty liposome group; (4) BLM + LPS + Clo group. The con empty liposome was prepared in PBS and the particle size was confirmed after filtering by 0.22 μm membrane. Mice in group (1)-(4) were first intratracheally injected with clodronate liposomes (1 mg/mL, 50 μl/mouse, YEASEN, Shanghai, China) or control empty liposome for 4 continuous days prior to IPF model building. All macrophage-depleted C57BL/6J mice were then injected BLM + LPS as above described. At the end of treatment, mice were anesthetized to test pulmonary functions and sacrificed to harvest lung samples for further experiments.

### 2.4 Bioinformatics analysis

The expression levels of SGK1 in lung tissues from patients with cirrhosis and healthy controls were acquired from the RNAseq dataset GSE118370 (n=12) and GSE98925 (n = 61) in the gene expression omnibus (GEO) database (www.ncbi.nlm.nih.gov/gds). Survival curves on the basis of SGK1 expression in lung cancer were constructed using the Kaplan-Meier method.

### 2.5 Pulmonary function detection

Flexivent system (Scireq, Montreal, Canada) was used to measure lung function as previously describe 5. Briefly, mice were intraperitoneally anesthetized with urethane (1.4 g/kg) and intraperitoneally injected with succinylcholine chloride (2 mg/kg) and then were intubated *via* a tracheostomy. After ventilated with medical grade oxygen, mice were connected to the Flexivent ventilator and assessed lung function with an automated measurement procedure three times for each mouse. Lung inspiratory capacity, resistance, lung compliance, elastance and total lung pressure-volume curves were then recorded.

### 2.6 Measurement of myeloperoxidase (MPO) and hydroxyproline (HYP)

After the mice were sacrificed, lung tissues were homogenized and lysed by RIPA and extracted the supernatant for following experiments. The contents of MPO and HYP in lungs were examed by their corresponding assay kits to evaluate the condition of fibrosis and inflammation, respectively.

### 2.7 Histological examination and immunohistochemistry

The lung samples were collected and then fixed with 4% formaldehyde solution, following embedded in paraffin and sliced into 5 μm tissue sections. Then the hematoxylin-eosin (H&E) and Masson's Trichrome staining were conducted as previously described. The Ashcroft scores were graded in 3 classes of increasing values: ranging from 0-3 (mild), 4 (moderate) and ≥ 5 (severe), and each lung sample were stained with Masson's Trichrome, and scored on a scale of 0-8 based on literature instruction 24. For IHC staining experiment, lung slices of human and mouse were prepared and incubated with primary antibody against SGK1 and further incubated with goat anti-mouse/rabbit IgG HRP polymer secondary antibody (ZSGB-BIO, Beijing, China). For quantitative assessment, three fields at least were randomly selected for each sample, and the area of the positive staining in was measured with ImagePro Plus 6.0 software.

### 2.8 Flow cytometry

Lung tissues of mice were collected after collagenase IV perfusion. The isolated lungs were cut into pieces and incubated in digestive solution with type IV collagenase for half 1 h at 37 °C. After filtered with a 70 μm cell strainer, cells were centrifuged at 1500 rpm for 10 min at 4°C. Then cell pellets were resuspended in the 1640 medium, stained with antibodies against CD3, CD4 and IL-17A in proper order and detected by the CytoFLEX flow cytometer (Beckman, USA).

### 2.9 Cell culture and treatment

Human myeloid leukemia mononuclear cells (THP-1) and human pulmonary fibroblasts (HPF) cells were obtained from ATCC. THP-1 cells were cultured in 1640 medium and HPF cells were cultured in Dulbecco's modified Eagle medium (DMEM), which all supplemented with 10 % (v/v) fetal bovine serum (FBS) (Gibco, 10099-141), penicillin (100 units/mL) and streptomycin (100 μg/ml). All cells were maintained in a humidified 5 % CO_2_ incubator at 37 °C. To induce macrophages polarization, THP-1 cells were treated with phorbol 12-myristate 13-acetate (PMA) for 2 days, and then treated with LPS (100 ng/mL) for 24 h to induce M1 macrophages or treated with IL-4 (20 ng/mL) for 24 h to induce M2 macrophages. For the SGK1 and TIP60 inhibition or the SGK1 activation studies, after 50 μM EMD and MG149-pretreated or 10 μM Dex-pretreated, THP-1 cells were further administrated with BLM, LPS or BLM+LPS for another 24 h. At the end of treatment, the conditional medium of THP-1 cells was collected and used for assays of western blot and transwell. The THP-1 and HPF cells were lysed for further experiments.

### 2.10 RNA sequencing

RNA-seq was conducted and sequencing libraries were built based on the Novaseq 6000 platform and the TruseqTM RNA sample prep kit (Illumina, San Diego, CA, USA). The total lung tissue RNAs was isolated with Trizol reagent, then the mRNA was divided using beads carried with oligo (DT) magnetic and shattered into small fragments randomly. 6-base random primers were used to reverse transcribe cDNA, followed by end repair and sequencing. The alternations in expressions of genes in lung samples following BLM, LPS or BLM + LPS administration were analyzed according to the identification of differently expressed genes (DEGs) (fold changes > 2 or < - 2; false discovery rate < 0.05). DEGs were clustered and analyzed using R software.

### 2.11 *In vitro* Th17 cell differentiation and migration assay

Naive CD4^+^ T cells were isolated using EasySep™ Mouse CD4^+^CD62L^+^ T Cell isolation Kit (Stemcell) and further incubated with anti-CD3 antibody (2.5 g/ml) and anti-CD28 antibody (5 g/ml) (Tonbo Biosciences). To induce Th17 cell differentiation, recombinant mouse IL-6 (50 ng/mL) and TGF-β (1 ng/mL) were added for 3 days. Cells were then seeded in top chambers of the transwell plates in FBS-free media. Then, 600 μl conditional medium of THP-1 treated with BLM, LPS or BLM + LPS were added to the bottom chambers of the transwell plates. After 24 h, the experiment was terminated by wiping the cells from the wells with a cotton swab, fixing with methyl alcohol for 15 min and staining with 0.1% crystal violet for 30 min. The photographic imaging was performed using an Olympus inverted microscope (40×) and the average number of migrated cells was calculated under the microscope for 5 views.

### 2.12 Immunofluorescence study

After treatment, cells and lung slices were fixed in 3.7 % formaldehyde, blocked and permeabilized with 1 % PBS-BSA containing 0.1 % Triton-X-100, followed by incubation with primary antibodies against fibronectin (FN), E-Cadherin, inducible nitric oxide synthase (iNOS), TGF-β1, mannose receptor (MR, CD206), alpha-smooth muscle actin (α-SMA), F4/80, CD4, interleukin 17A (IL-17A) and macrophage inflammatory protein 1gamma (MIP-1γ) at 4 °C overnight. The next day, samples were incubated with Alexa Fluor® 488 Goat anti-mouse secondary antibody (dilution, 1:1000) or Alexa Fluor® 594 Goat anti-rat secondary antibody (dilution, 1:1000) and then counterstained with DAPI. All images were collected with confocal laser scanning microscope (#LSM800, Leica, JPN) at 40× magnification, and fluorescence intensities of target proteins were calculated using ImageJ software.

### 2.13 Quantitative real-time PCR

Total RNA was obtained from mice lungs by Fast Pure Cell/Tissue Total RNA isolation kit (RC101-01) from Vazyme Biotech (Nanjing, China). The RNA concentrations were detected and reverse transcribed into cDNA, which was then used for real-time PCR analyses with SYBR Green Master Mix (Q511-02, purchased from Vazyme Biotech) and specific primers. The results were calculated and then normalized with *Hprt1*.

### 2.14 Western Blot analysis

The total proteins were obtained from lung tissues of mice and cells using RIPA buffer purchased from Beyotime Institute of Biotechnology (Shanghai, China). Equivalent protein was resolved on 10 % SDS-PAGE gel, transferred to PVDF membranes (Merck Millipore, Darmstadt, Germany) and cultivated with different primary antibodies overnight. Bands were incubated with relative secondary antibodies for 1 h and imaged by ChemiDocTM Touch Imaging System (Bio-Rad, Hercules, CA) after being washed with TBST buffer.

### 2.15 Chromatin immunoprecipitation (ChIP)-qPCR

Samples were collected and processed into cross-linked digested chromatin using thermo Pierce Agarose ChIP Kit (Thermo Scientific), and then immunoprecipitated with H3K27ac antibody except for the control beads and rotated at 4°C for 1 h. Then samples were supplemented with protein occluded A/G microspheres and spun at 4°C overnight. The eluted DNA were further treated with proteinase K. Subsequently, the DNA was purified using the qPCR purification kit. Primers were designed to target different regions of interest genes for the following gene expression assessment by q-PCR. The fold enrichment of target genes of IP-DNA and input DNA were determined by control group.

### 2.16 Statistical analysis

All experiments were conducted at least three times independently and depicted by mean ± SD. The differences among different groups were compared by One-way ANOVA analysis using GraphPad Prism 8. The significance of statistics was performed using: *P < 0.05; **P < 0.01; ***P < 0.001.

## 3. Results

### 3.1 SGK1 expression is upregulated in IPF patient lungs and is closely related to the polarization of M2 macrophages

**Figure 1:**
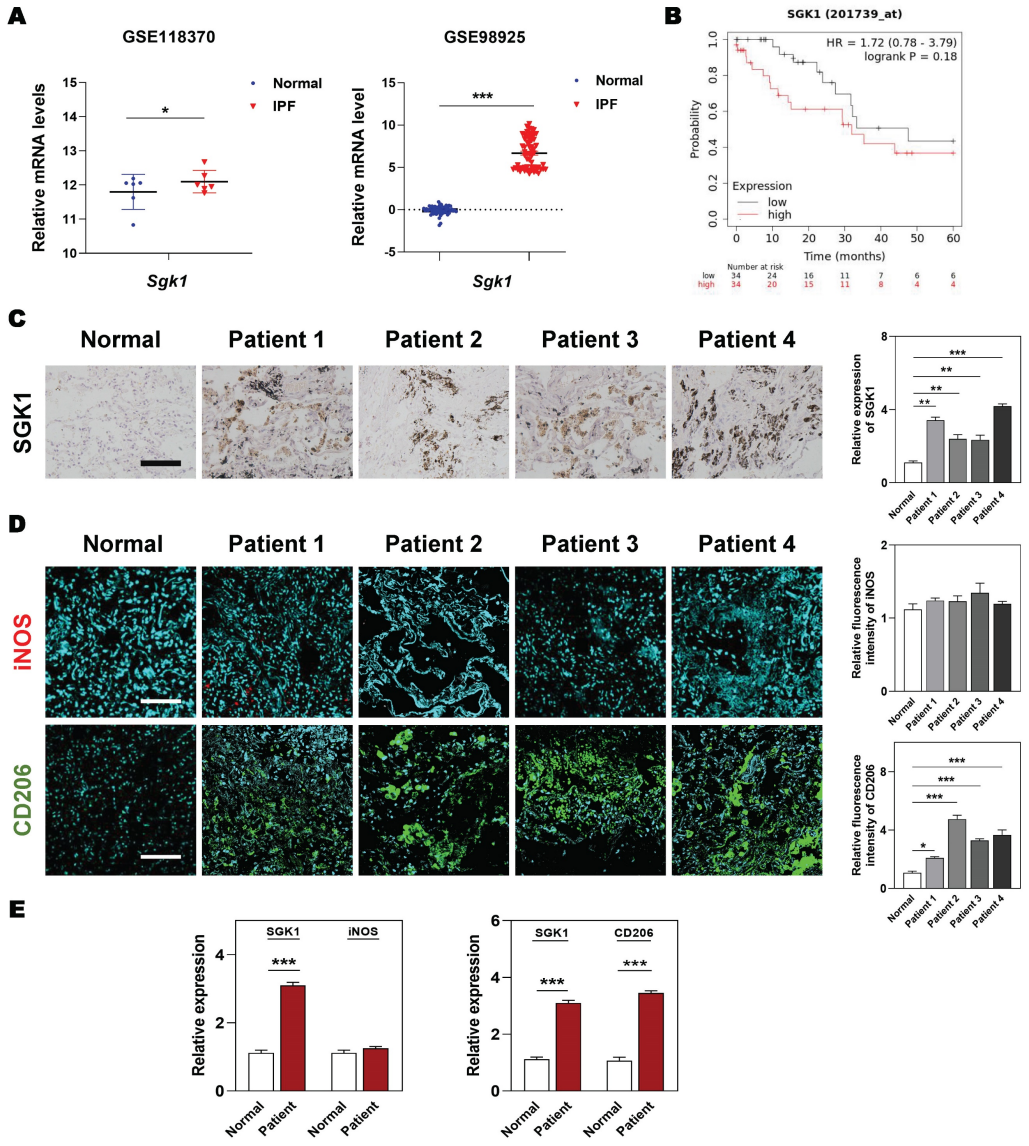
** Enhanced SGK1 in IPF patients is correlated with the reprograming of M1 to M2 macrophages. (A)** The mRNA levels of *Sgk1* in healthy controls and IPF patients cultivated from GEO databases. **(B)** The overall survival of patients with low and high SGK1 expression. **(C-E)** The selection and source of patient samples are shown in **Table S1**. **(C)** Representative images of immunohistochemistry staining in normal and patient lungs. Scale bar = 100 µm. **(D)** Representative images of immunofluorescent staining of iNOS and CD206 in normal and patient lungs. Scale bar = 200 µm. **(E)** The quantification of SGK1, iNOS and CD206 expression. Statistical significance: *P < 0.05, **P < 0.01, ***P < 0.001, compared with relative groups (n = 6).

At the beginning, we wanted to clarified the clinicopathological and prognostic significance of SGK1 in lung diseases. Based on the clinical genomic data of GEO datasets (GSE118370 and GSE98925), the expression of SGK1 was observed to be dramatically increased in the patients with IPF compared with the healthy controls (**Figure 1A**). Of note, the overall survival is significantly lower in SGK1^high^ patients with lung cancers who experiences IPF symptoms, when compared with SGK1^low^ individual (**Figure 1B**). To further evaluate the potential association of SGK1 with IPF, we analyzed the expression of SGK1 in lung tissues from 27 patients with chronic inflammation and pulmonary fibrosis who underwent lung surgery (the baseline characteristics of the IPF patients are shown in **Table S1**). IHC staining further showed that SGK1 expression was significantly increased in the lungs of different pulmonary fibrosis patients compared with healthy controls (**Figure 1C**). These results preliminarily suggest that a positive correlation may exist between SGK1 and IPF progression. To further verify our conjecture, we constructed acute and chronic IPF mouse models and performed PCR and IHC analysis to check SGK1 expression. As a result, the mRNA expression (**Figure S1A**) and immunoreactivity (**Figure S1B**) of SGK1 were increased from normal mouse lungs to acute (not significant) and further chronic IPF (significant) in a stepwise manner in the BLM + LPS group. Considering the important role of macrophages played in pulmonary fibrosis 25, we further explored whether the high expression of SGK1 was related to any phenotype changes of macrophage. Notably, immunofluorescent images showed that the expression of CD206 (a marker of M2 macrophage) rather than iNOS (a marker of M1 macrophage) had a significant increase in IPF patients compared with normal group (**Figure 1D** and** S1C**) and was positively correlated with SGK1 expression (**Figure 1E**). These findings indicated that SGK1 might be a crucial marker of IPF and closely related to the polarization of M2 macrophages.

### 3.2 SGK1 participates in the progression of BLM + LPS-induced pulmonary fibrosis in mice

**Figure 2:**
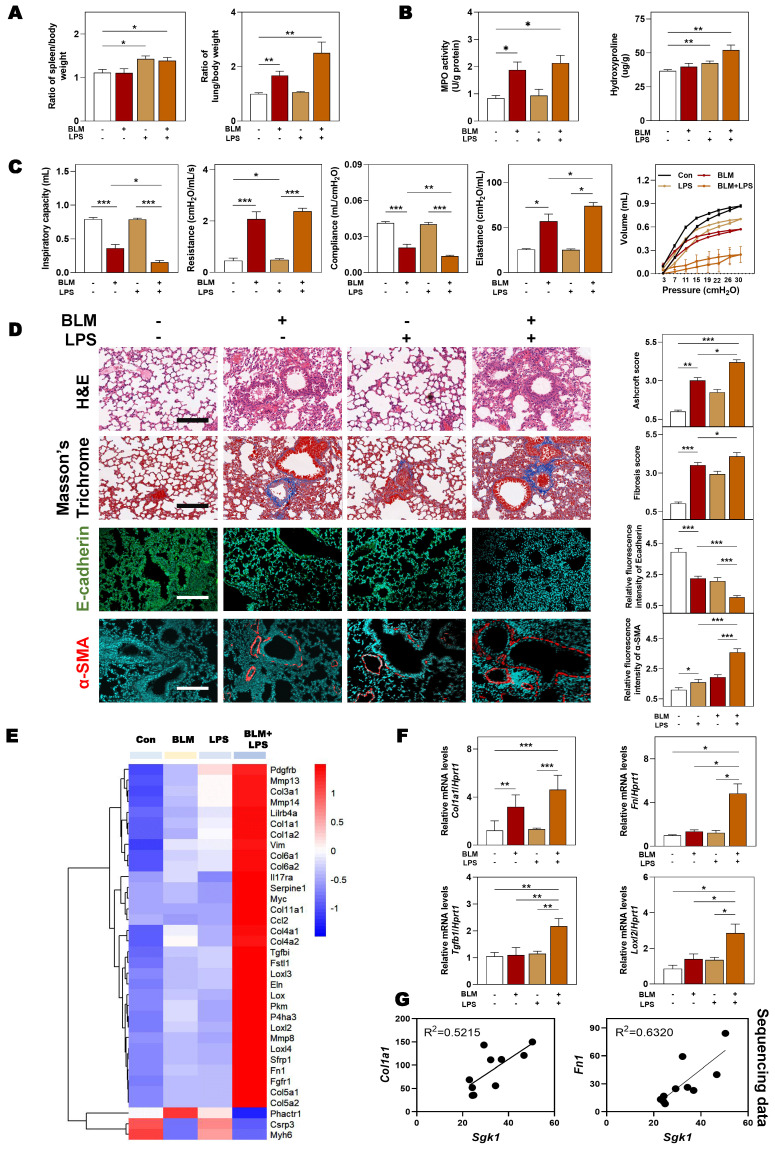
** SGK1 drives the proinflammatory and fibrotic injury in BLM plus LPS-induced pulmonary fibrosis mouse model.** Mice were received with BLM (2.5 mg/kg), LPS (1 mg/kg) or BLM + LPS by intratracheal instillation as described in the **Methods**. **(A)** Ratio of spleen and lung to body weight. **(B)** The MPO and HYP levels in lungs. **(C)** Lung inspiratory capacity, resistance, compliance, elastance and total lung pressure-volume curves. **(D)** Representative images of H&E, Masson's trichrome staining (Scale bar = 100 µm) and immunofluorescent staining of E-cadherin and α-SMA in lungs (Scale bar = 200 µm). **(E)** RNA sequencing data of differentially expressed genes in lungs. **(F)** Relative mRNA levels of *Col1a1*, *Fn*, *Tgfb1* and *Loxl2* were determined by qPCR and normalized using *Hprt1* as an internal control. **(G)** The correlation analysis of *Sgk1* with *Col1a1* and *Fn1*. Statistical significance: *P < 0.05, **P < 0.01, ***P < 0.001, compared with relative groups (n = 6).

Based on the above observations, we next sought to clarify that our animal model could accurately reflect the pathological changes of clinical IPF patients before we demonstrated the relationship between SGK1 and lung fibrosis. The significant increased ratios of spleen and lung weight over body weight caused by BLM + LPS were observed (**Figure 2A**). Compared with Con group, the content of myeloperoxidase (MPO, a marker of inflammation and oxidative processes) was markedly increased in the BLM and BLM + LPS group and the level of hydroxyproline (HYP, a marker of fibrosis) was markedly upregulated in the LPS and BLM + LPS group (**Figure 2B**). In addition, the pressure-volume curves in BLM plus LPS- treated mice revealed a more severe right-downward shift, corresponding to an increase of lung resistance and elastance as well as a reduction in lung inspiratory capacity and compliance compared with the Con, BLM or LPS group (**Figure 2C**). Specifically, H&E and Masson's trichrome staining indicated that inflammatory cell infiltration, destroyed lung architecture and collagen fiber depositions were more severe in BLM + LPS group compared with BLM or LPS group. Also, the expression of E-cadherin (a marker of adherent junction protein responsible for maintaining tissue structure) was significantly decreased but the expression of α-SMA was markedly increased in the BLM + LPS group compared with BLM or LPS group (**Figure 2D**). Subsequently, transcriptome sequencing was used to further analyze the deeper mechanism of BLM- and LPS-induced pulmonary fibrosis. Consistently, the sequencing analysis indicated the significantly increased expression of fibrogenic markers such as collagen type 1 alpha 1 (*Col1a1*), *Tgfb1*, *lysyl oxidase like-2* (*Loxl2*) and *fibronectin* (*Fn1*) and the decreased expression of muscle homeostasis-related genes such as phosphatase and actin regulator 1 (*Phactr1*), cysteine and glycine-rich protein 3 (*Csrp3*) and myosin heavy chain 6 (*Myh6*) in the lungs of BLM + LPS group (**Figure 2E**). We further determined the mRNA levels of related fibrotic markers in the lung homogenates, and confirmed that the mRNA level of *Col1a1*, *Fn*, *Tgfb1* and *Loxl2* in the BLM + LPS group were much upper than that in the Con group or group treated with BLM or LPS alone (**Figure 2F**). Notably, we also confirmed the positive correlations of *Sgk1* with typical fibrotic markers (*Col1a1* and *Fn1*) as expected (**Figure 2G**). These results suggested that SGK1 participated in the process of BLM + LPS-induced pulmonary fibrosis in mice.

### 3.3 Highly-expressed SGK1 is accompanied by reprogramming of macrophages from M1 to M2 state

**Figure 3:**
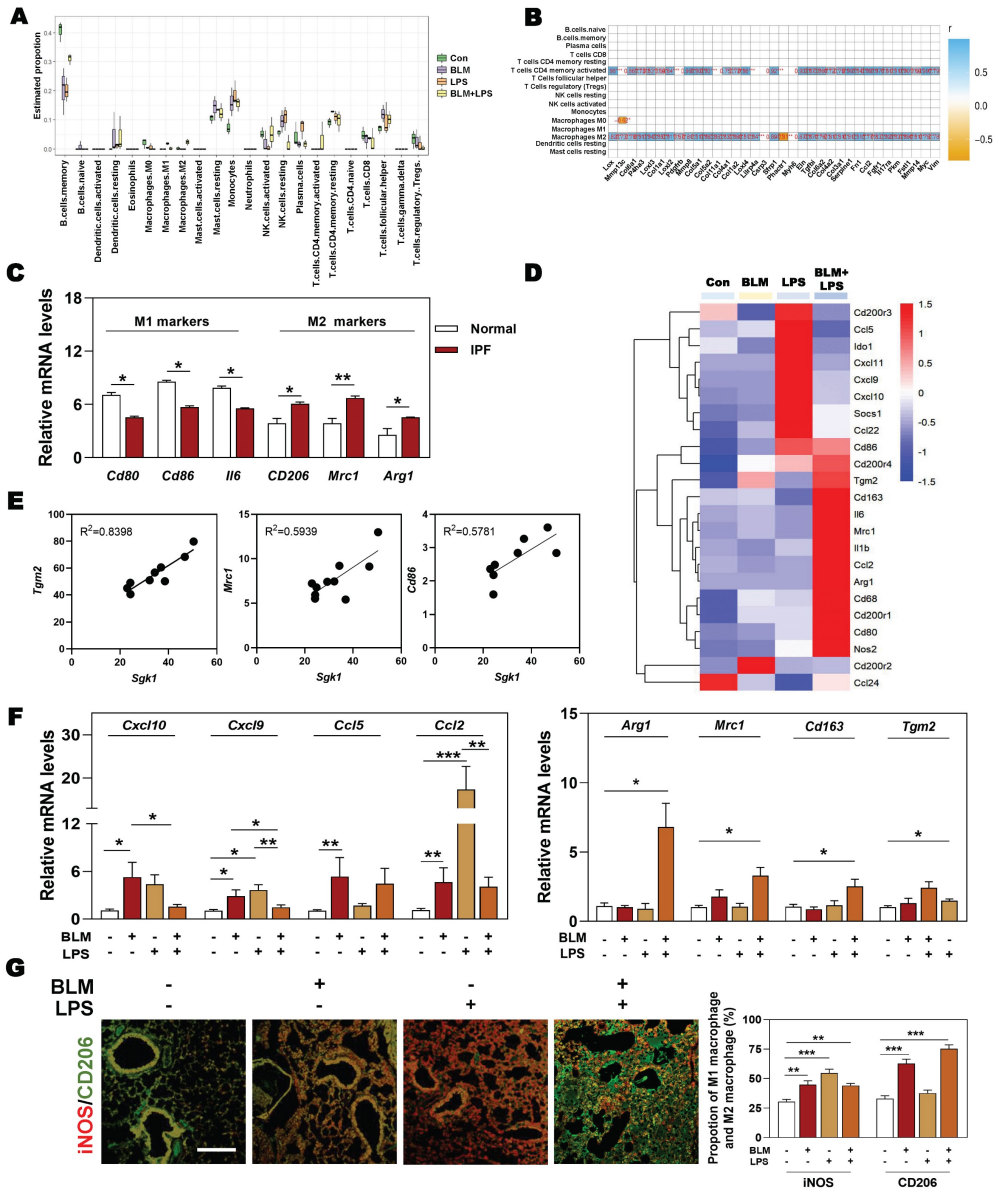
** The upregulation of SGK1 is closely correlated with reprogramming of M2-type macrophages. (A** and** B)** The correlation analysis of differentially expressed genes with different types of immune cells in lungs. **(C)** Relative mRNA levels of M1 markers (*Cd80*, *Cd86* and *Il6*) and M2 markers (*Cd206*, *Mrc1* and *Arg1*) in GEO database.** (D)** The expression of M1 and M2 macrophage-associated significantly changed genes in lung sequencing data (n = 4). **(E)** The correlation analysis of* Sgk1* with *Tgm2*, *Mrc1* and *Cd86*. **(F)** The mRNA levels of *Cxcl10, Cxcl9, Ccl5*,* Ccl2*, *Arg1*, *Mrc1*, *Cd163* and *Tgm2* were determined by qPCR and normalized using *Hprt1* as an internal control. **(G)** Representative images of immunofluorescent staining of iNOS and CD206 in lungs. Scale bar = 200 µm. Statistical significance: *P < 0.05, **P < 0.01, ***P < 0.001, compared with relative groups (n = 6).

Next, we wondered whether there was a relationship among SGK1-associated IPF and different types of immune cells, especially for macrophages. The results of correlation analysis showed that macrophages and T cells were the significantly altered cell types in different groups (**Figure 3A**). Specifically, we also found that the markedly upregulated fibrotic genes were strongly correlated with the increased population of M2 macrophages and CD4^+^ T cells (**Figure 3B**). Consistently, the clinical GEO results showed that M2 markers rather than M1 markers were highly expressed in patients with IPF (**Figure 3C**). Our sequencing data also revealed that the expression level of M2 markers such as *Cd86*, mannose receptor C-type 1 (*Mrc1*) and arginase1 (*Arg1*), rather than M1 markers such as CD200 receptor 3 (*Cd200r3*), chemokine (C-X-C motif) ligand 9 (*Cxcl9*) and *Cxcl10*, were significantly increased in the BLM + LPS group, when compared with the Con group (**Figure 3D**). Our qPCR results further confirmed that the expression of M2-type macrophage markers like *Arg1*, *Mrc1*, *Cd163* and transglutaminase 2 (*Tgm2*) were significantly upregulated in the BLM + LPS group (**Figure 3F**). Again, as expected, the correlation analysis between SGK1 and M1/M2 (based on RNA-sequence data) showed that the expression of *Sgk1* was positively correlated with the expression of M2 macrophage markers (**Figure 3E**). In particular, there was an interesting transition between M1 and M2 after observing that these mentioned M2 markers were significantly increased while M1 markers (*Cxcl10*, *Cxcl9*, *Ccl5* and *Ccl2*) were markedly or slightly decreased after BLM + LPS intervention compared with BLM or LPS alone treated groups (**Figure 3F**). Similar results of the co-immunostaining analysis also showed that M2-type macrophages were the predominant cells in the lungs of BLM + LPS challenged mice as companied by the decrease of M1 macrophages expression (as indicated by the significantly increased green fluorescent area in BLM + LPS group) (**Figure 3G** and **Figure S2**). Collectively, these results suggested that BLM + LPS-induced lung injury might promote the transition of M1 to M2 macrophages and SGK1 is probably capable of ensuring this process.

### 3.4 SGK1-enriched M2 macrophages release TGF-β to further activate fibroblasts and accelerate fibrotic progression

**Figure 4:**
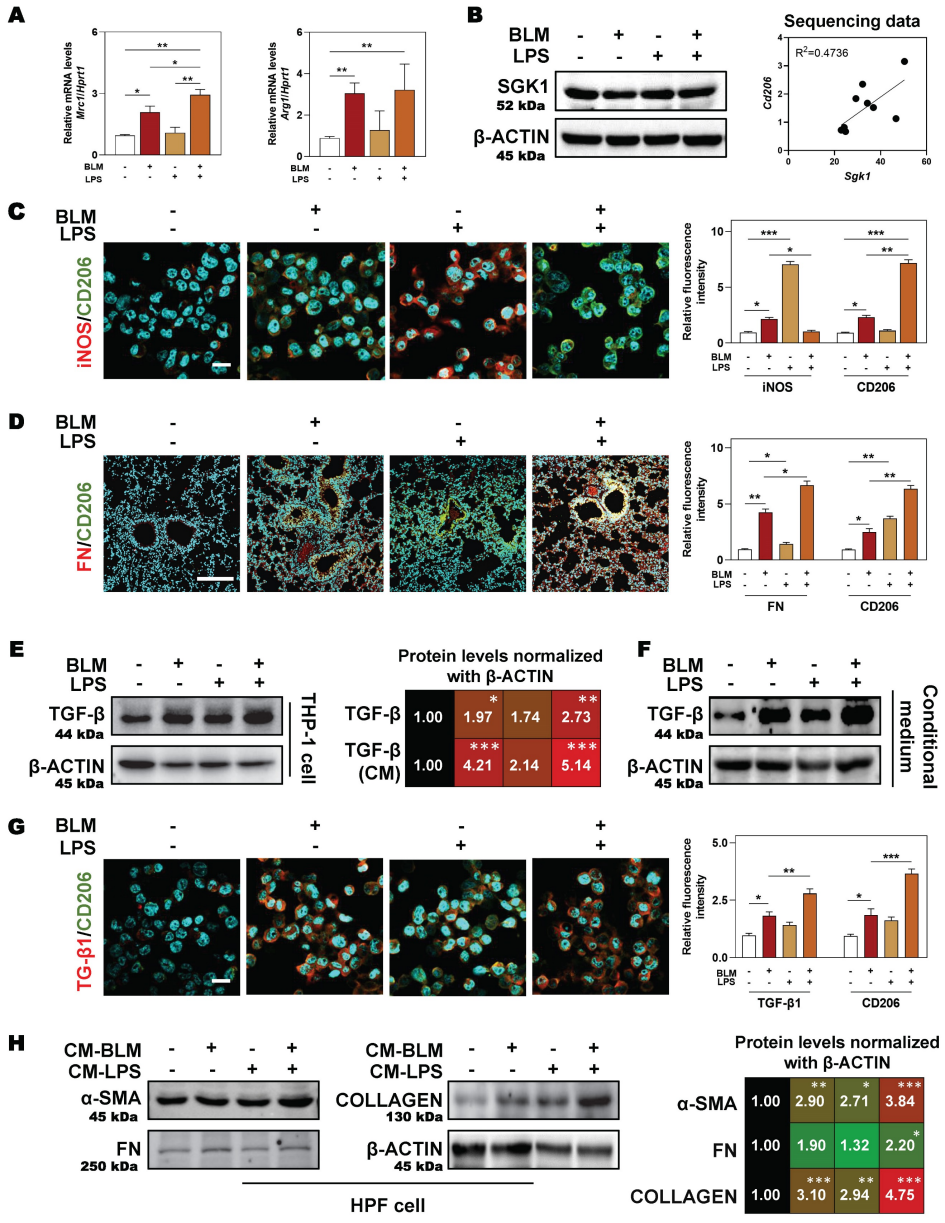
** SGK1-enriched M2 macrophages promotes HPF activation by releasing TGF-β.** THP-1 cells were pre-treated with PMA for 2 days and then treated with BLM (50 μM), LPS (100 ng/mL) or BLM + LPS for another 24 h, and the conditional medium was collected. HPF cells were treated with relative medium for 24 h (details listed in **Methods**). **(A)** Relative mRNA levels of *Mrc1* and *Arg1* in THP-1 cells were determined by qPCR and normalized using *Hprt1* as an internal control. **(B)** Representative immunoblots against SGK1 and β-ACTIN in THP-1 cells** (left panel)**. The correlation analysis of *Sgk1* with *Cd206* based on our sequencing data was shown **(right panel)**. Representative images of immunofluorescent co-staining of iNOS and CD206 **(C)** in THP-1 cells (Scale bar = 50 µm), and co-staining of FN and CD206 **(D)** in lung tissues (Scale bar = 200 µm).** (E-F)** Representative immunoblots against TGF-β and β-ACTIN in THP-1 cells and their conditional medium were shown, respectively. **(G)** Representative images of immunofluorescent result of TGF-β and CD206 in THP-1 cells treated with BLM and/or LPS. Scale bar = 50 µm.** (H)** Representative immunoblots against α-SMA, FN, COLLAGEN and β-ACTIN in HPF cells treated with conditional medium (CM) from relative macrophages. Statistical significance: *P < 0.05, **P < 0.01, ***P < 0.001, compared with relative groups (n = 3).

Expect for clinical samples and mouse lungs, BLM plus LPS also activated SGK1 expression and promoted the transition from M1 to M2 state in THP-1 cells (human monocyte cell line that has been extensively used to study macrophage functions), as evidenced by the comparable PCR results (higher level of* Mrc1* and *Arg1*) (**Figure 4A**), protein expression (upregulated SGK1 expression, **Figure 4B**, **left panel**), positive correlation of *Sgk1* and M2 marker *Cd206* based on our RNA sequencing data (**Figure 4B**, **right panel**) and immunofluorescence staining results (increased staining area of CD206 but not iNOS (**Figure 4C** and **Figure S3A**). We then investigated whether these Sgk1-enriched M2 macrophages promoted fibrosis and found that the combination of BLM and LPS greatly increased the expression of FN around macrophages *in vivo* (**Figure 4D** and **Figure S3B**). Herein, we further examined different stimulators for the progression of pulmonary fibrosis and found that there was a markedly increased expression of TGF-β both in the BLM- and BLM + LPS-treated THP-1 cells (**Figure 4E**, **4G** and **Figure S3C**) and even in the treated THP-1-derived conditional medium (**Figure 4F**). Thus, we speculated that BLM and LPS promoted the M2 transformation of macrophages and the release of TGF-β from alternatively activated macrophages further affect the fate of surrounding cells. Later, this hypothesis was confirmed by the upregulated fibrotic markers including α-SMA, FN and COLLAGEN in HPF cells (human pulmonary fibroblasts cell line) treated with THP-1 conditional medium (**Figure 4H**). Together, our data suggest that BLM plus LPS increased the number of M2 macrophages along with increased TGF-β1 secretion, thereby activating HPF cells, further inducing them to differentiate into myofibroblasts and driving the fibrosis chain.

### 3.5 SGK1 is related to the recruitment of Th17 cells by secreting CCL9 and aggravates fibrosis injury

**Figure 5:**
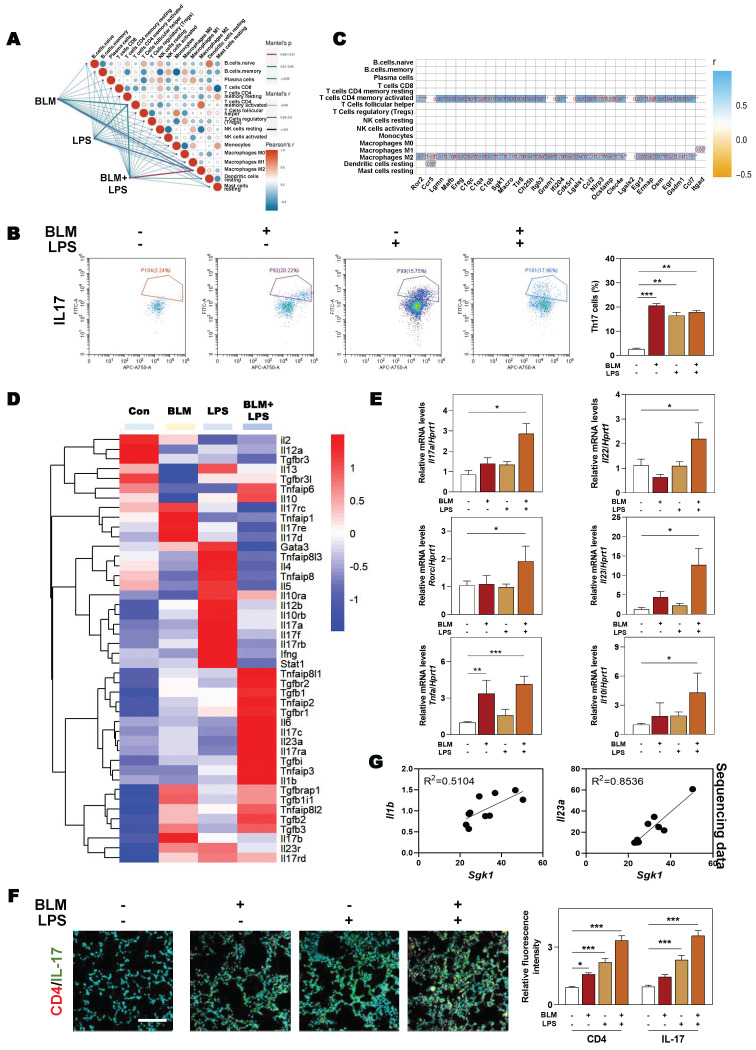
** SGK1 promotes the recruitment of Th17 cells and aggravates fibrosis injury. (A)** The difference of genes involved in the immune cells between the different groups of administration (BLM (2.5 mg/kg) and LPS (1 mg/kg). **(B)** Flow cytometry analyzes number of Th17 (CD3^+^+CD4^+^+IL17^+^) cells in the lung. **(C)** The correlation analysis of DEGs with different types of immune cells.** (D)** The RNA sequencing data of DEGs related to inflammatory reaction during IPF. **(E)** The mRNA expression of *Il17a, Il22, Rorc*, *Il23*, *Tnfα* and *Il10* in lung tissue and normalized to *Hprt1*. **(F)** Representative images of immunofluorescent result of CD4 and IL-17 in lungs. Scale bar = 200 µm. **(G)** The correlation analysis of *Sgk1* with *Il-1b* and *Il-23a*. Statistical significance: *P < 0.05, **P < 0.01, ***P < 0.001, compared with relative groups (n = 6).

Our data in **Figure 3B** also revealed that apart form M2 macrophages, CD4^+^ T cells were also strongly correlated with the significantly changed genes involved in fibrotic process. Therefore, we next examined the changes and relevance of different immune cells and our correlation results showed that there was a positive correlation between macrophage and CD4^+^ T cell after BLM and LPS administration (**Figure 5A**). Generally, among numerous subsets of CD4^+^ T cells, Th17 cells are commonly known as proinflammatory cells and characterized by conventional Th17 molecule expression including retinoid-related orphan receptor-gamma t (RORγt) and the production of cytokines such as Tnfα, IL-17, IL-22 and IL-23 4. We further isolated different immune cells and observed that BLM + LPS mainly affected the recruitment of Th17 cells rather than other T cells such as Th1 cells, Th2 cells or Treg cells, as determined by flow cytometry analysis (**Figure 5B** and **Figure S4A**). In addition, our correlation analysis between immune cells and the significantly changed genes in our sequencing data further pointed out the importance of Th17 cells played in the progression of pulmonary fibrosis (**Figure 5C**). Multiple immune cells express specific cell membrane markers and secrete different and specific cytokines or chemokines to participate in the dynamic balance of the immune microenvironment. As depicted in **Figure 5D**, the cytokines released by Th17 cells such as *Il17c*, *Il23a* and *Il10* were dramatically upregulated while the expression of anti-inflammatory cytokines (*Il4* and *Il5*) and several transcription factors (GATA binding protein 3 (*Gata3*) and signal transducer and activator of transcription 1 (*Stat1*) were markedly decreased in the BLM + LPS group, when compared with other groups according to our sequencing data. Also, PCR results showed that the expressions of cytokines including *Il17a*, *Il22*, *Il23* and *Il10* as well as *Rorc* and *Tnfa* were significantly elevated after BLM + LPS administration compared with the Con group in lung tissue (**Figure 5E**). Meanwhile, the increase of Th17 cells caused by BLM plus LPS was also verified by the co-immunostaining of CD4 and IL-17 (a typical cytokine of Th17 cells) (**Figure 5F** and **Figure S4B**). Interestingly, after in-depth analysis of sequencing data, we found a positive correlation between *Sgk1* and the cytokines related to Th17 cells, such as *Il1b* and *Il23a* (**Figure 5G**). Collectively, our results indicated that the number and function of Th17 cells were significantly enhanced after treated with BLM plus LPS and were strongly correlated with SGK1 in the lung.

**Figure 6:**
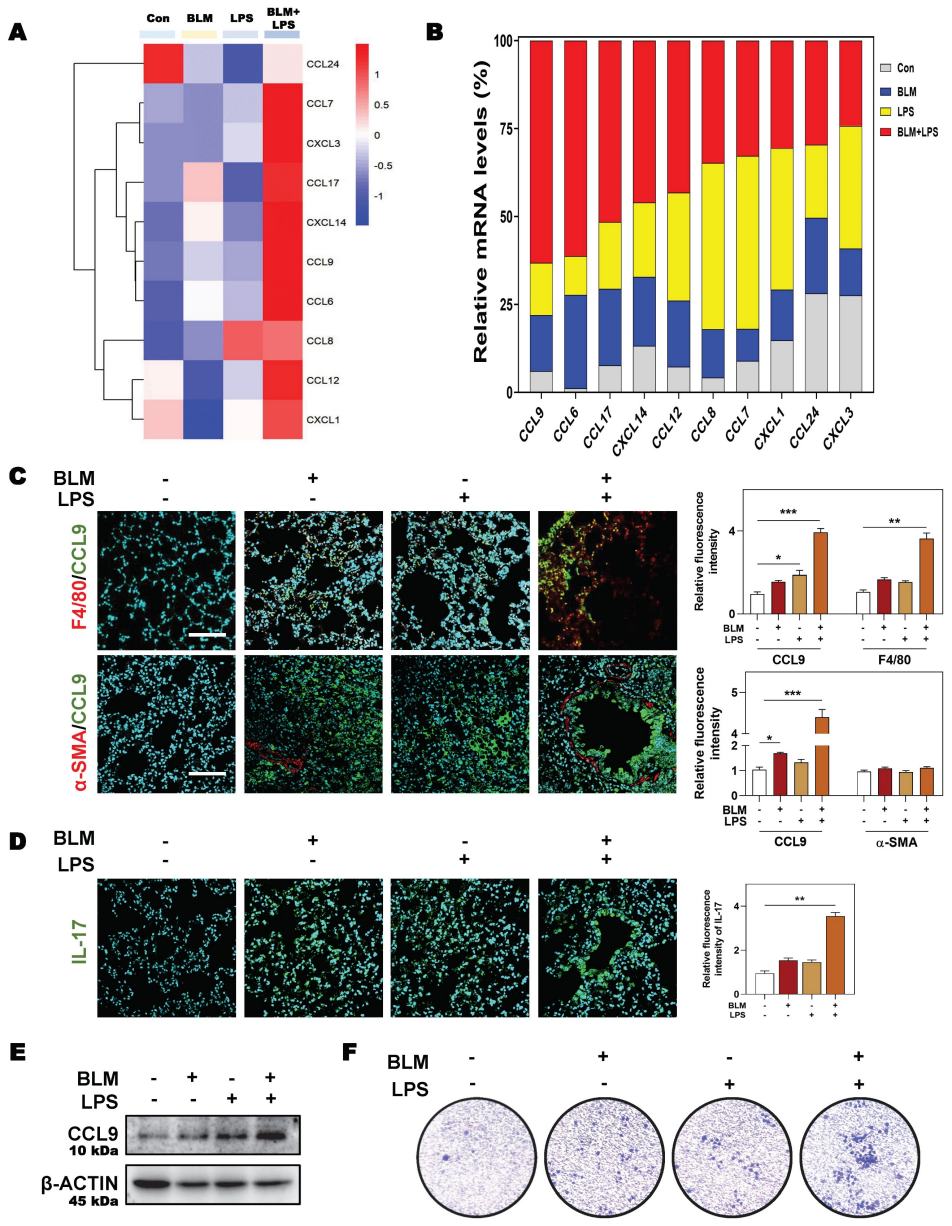
** SGK1 induces the activation of Th17 cells by promoting M2 macrophages reprogramming and then the secretion of CCL9. (A** and** B)** The RNA sequencing data and proportion of chemokine-related differential gene expression. Representative images of co-immunofluorescent staining of CCL9 with F4/80 and α-SMA **(C)** and the expression of IL-17 **(D)** in lungs treated with BLM and/or LPS. Scale bar = 200 µm. **(E)** Representative immunoblots against CCL9 and β-ACTIN in THP-1 cells treated with BLM and/or LPS. **(F)** Representative images of Th17 cells migration assay. Statistical significance: *P < 0.05, **P < 0.01, ***P < 0.001, compared with relative groups (n = 3).

Considering the possible way that macrophages disturbing immune cells network, we then analyzed the expression of different chemokines in fibrotic lung tissue. Multiple chemokines such as *Ccl9*, *Ccl6*, *Ccl7*, *Ccl17*, *Cxcl3* and *Cxcl14* were all significantly elevated in BLM + LPS-challenged mice compared with other groups (**Figure 6A**). Notably, among these chemokines, CCL9 was found to be the most upregulated factor by comparing the increase rate of different chemokines in fibrotic lungs than in normal lungs as proved by qPCR assay (**Figure 6B**). To identify the cellular source of endogenous CCL9 in fibrotic lung tissues, we examined the colocalization of CCL9 with F4/80 (macrophages marker) and α-SMA (myofibroblasts marker) in mouse fibrotic lung tissues by immunofluorescence staining. CCL9 significantly co-localized with F4/80 but did not largely co-localize with α-SMA, suggesting that macrophage rather than the myofibroblasts were the main producers of CCL9 in fibrotic lung tissues (**Figure 6C** and **Figure S5**). Moreover, immunostaining analysis showed strong expression of IL-17 in lung tissues obtained from BLM-, LPS- or BLM + LPS-treated IPF mice than controls (**Figure 6D**). Then we wanted to determine whether macrophages release CCL9, which in turn affect Th17 recruitment. We detected the increased CCL9 level in conditional medium of BLM, LPS and especially, BLM plus LPS-treated THP-1 cells (**Figure 6E**). Also, the transwell migration assay showed that BLM + LPS challenged THP-1 cells exhibited a much stronger effects on Th17 cell migration compared with other groups (**Figure 6F**). These data suggested that M2-type macrophages secreted CCL9 to recruit and further activate Th17 cells in the lung, promoting IPF progression.

### 3.6 SGK1 activates the GSK3β-TIP60 cascade to trigger the acetylation of H3K27 and promotes the expression and secretion of TGF-β and CCL9

**Figure 7:**
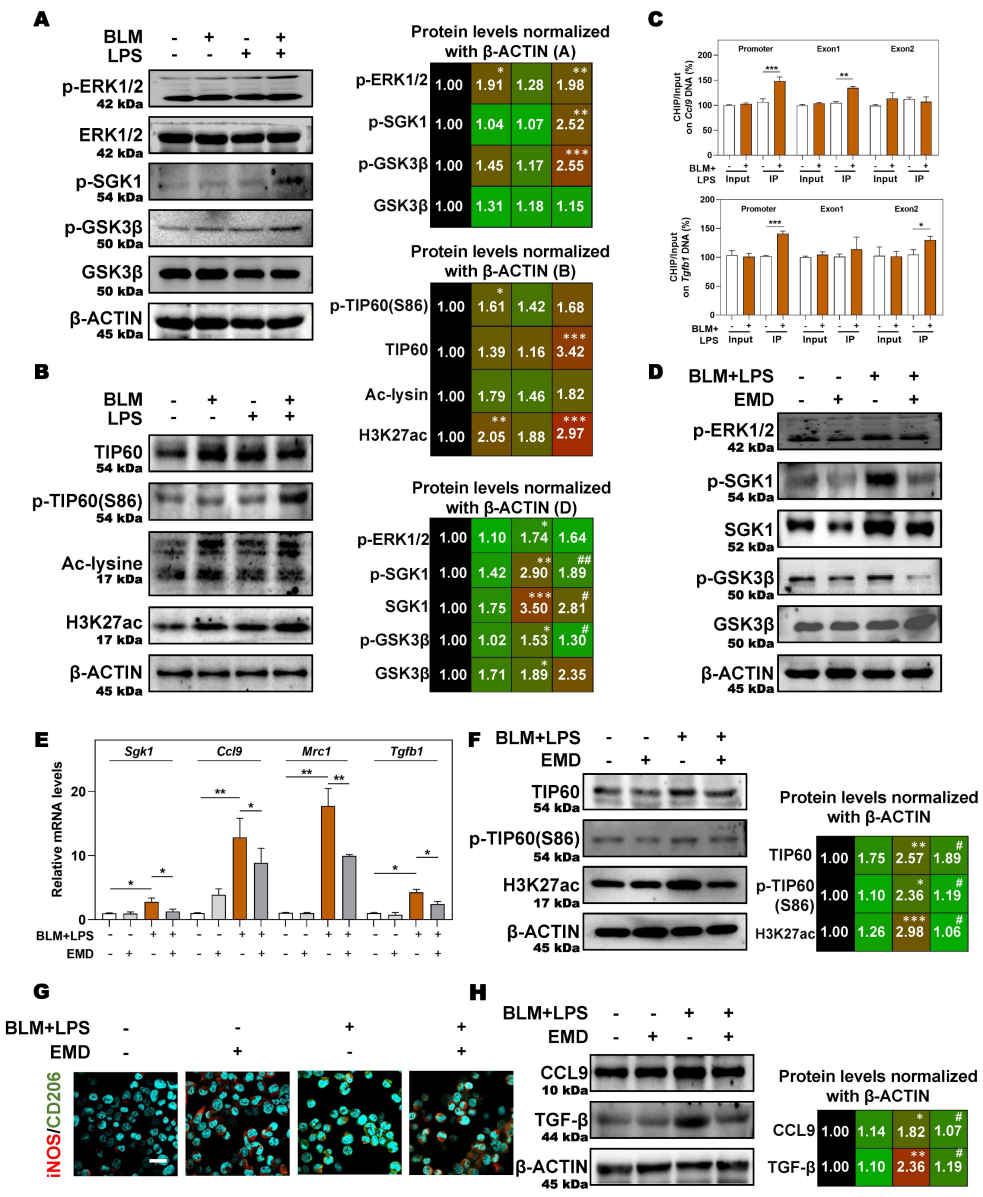
**SGK1 activates the GSK3β-TIP60 cascade to trigger the acetylation of H3K27. (A** and** B)** THP-1 cells were pre-treated with PMA for 2 days and then treated with BLM, LPS or BLM + LPS for another 24 h. After pre-treated with PMA for 2 days, THP-1 cells were administrated with EMD **(D-H)** for 1 h and then treated with BLM + LPS for another 24 h. **(A, B, D, F** and** H)** Representative immunoblots against p-ERK1/2, ERK1/2, p-SGK1, SGK1, p-GSKβ, GSK3β, TIP60, p-TIP60 (S86), Ac-lysine, H3K27ac, CCL9, TGF-β and β-ACTIN in THP-1 cells. **(C)** Input normalized H3K27ac enrichment levels were determined by ChIP-qPCR in the regions of promoter, exon1 and exon2 of *Ccl9* (upper side) and *Tgfb1* (lower side). **(E)** The mRNA expression of *Sgk1, Ccl9, Mrc1* and *Tgfb1* and normalized to *Hprt1* mRNA levels. **(G)** Representative images of immunofluorescent result of iNOS and CD206 in THP-1 cells. Scale bar = 50 µm. Statistical significance: *P < 0.05, **P < 0.01, ***P < 0.001, compared with control group; ^#^*P* < 0.05, ^##^*P* < 0.01, compared with BLM + LPS group (n = 3).

Next, we aimed to further clarify the specific mechanisms by which SGK1 induced the reprogramming of M2 macrophages and induced the secretion of TGF-β and CCL9. SGK1 displays serine/threonine kinase activity and can also be phosphorylated by several distinct protein kinases 26. We therefore analyzed the phosphorylation of extracellular signal-regulated kinase 1/2 (ERK1/2), GSK3β and other kinases known to be involved in the regulation of protein synthesis by immunoblotting. We found that the protein expression of ERK1/2 phosphorylation, p-SGK1 and p-GSK3β were enhanced in THP-1 cells in response to BLM + LPS challenge, while the abundance of ERK1/2 and GSK3β was unchanged (**Figure 7A**). Considering that SGK1 could not directly affected the transcription level of M2 marker genes, thus we wondered whether there are several other regulation approaches and then studied the direct effect of SGK1-GSK3β on different epigenetic pathways *in vitro*. We found that the activated SGK1 by BLM + LPS mainly affected the process of histone acetylation, rather than other modification process such as histone methylation and ubiquitination.

Our data revealed that the expressions of classic acetyltransferase TIP60, TIP60 (S86), who acetylated proteins Ac-lysine, and the key histone modification protein H3K27ac were both significantly increased after BLM + LPS insult, whereas the expression of other classic acetyltransferases CBP-associated factor (PCAF) and p300, and another histone modification protein, such as H3K9ac was not altered in the THP-1 cells treated with BLM and LPS (**Figure 7B** and **Figure S6A**). Then, we designed specific primers at the promoter and exon regions of TGF-β and CCL9 to determine the occupation of H3K27ac modification at these gene locus. As shown in **Figure 7C**, H3K27ac was markedly upregulated at the promoters and exon1 regions of *Ccl9* as well as the promoter and exon2 regions of *Tgfb1* in BLM + LPS-treated groups.

In addition, in the presence of the SGK1 inhibitor EMD 27, the increased expression of p-ERK1/2 was almost unchanged but the phosphorylation of SGK1, SGK1 and p-GSK3β were decreased, which indicated that GSK3β phosphorylation might be under the control of SGK1 (**Figure 7D** and **Figure S6B**). Meanwhile, if cells were pre-treated with MG149 (TIP60 inhibitor), the expressions of p-ERK1/2, ERK1/2, p-GSK3β and GSK3β were almost unchanged while the phosphorylation levels of SGK1 and TIP60 (S86) were significantly decreased, when compared with BLM + LPS group (**Figure S6C**), indicating that TIP60 was the downstream of GSK3β signaling. Besides, the upregulated mRNA expressions of *Sgk1*,* Ccl9*, *Mrc1* and *Tgfb* (**Figure 7E**), the increased protein levels of TIP60, p-TIP60 (S86), and H3K27ac (**Figure 7F**), the increased ratio of stained area of M2 marker to M1 marker (**Figure 7G** and **Figure S6D**) in THP-1 cells, and the enhanced release of CCL9 and TGF-β (**Figure 7H**) in the conditional medium of THP-1 cells treated with BLM plus LPS were all reversed by SGK1 inhibitor, EMD. Meanwhile, the upregulated SGK1, TIP60, p-TIP60 (S86), and the increased ratio of M2 stained areas to M1 stained areas caused by BLM+LPS, as well as the enhanced release of CCL9 and TGFβ in the conditional medium of THP-1 cells after BLM+LPS administration, were further enhanced by activating SGK1 (**Figure S7A** and** S7B**). Taken together, our results suggested that macrophage reprogramming and subsequent fibrotic pulmonary injury were regulated by SGK1-mediated GSK3β-TIP60-H3K27ac cascade, which promoted the transcription and secretion of TGF-β and CCL9.

### 3.7 Depletion of macrophages partly eliminates SGK1-triggered IPF

**Figure 8:**
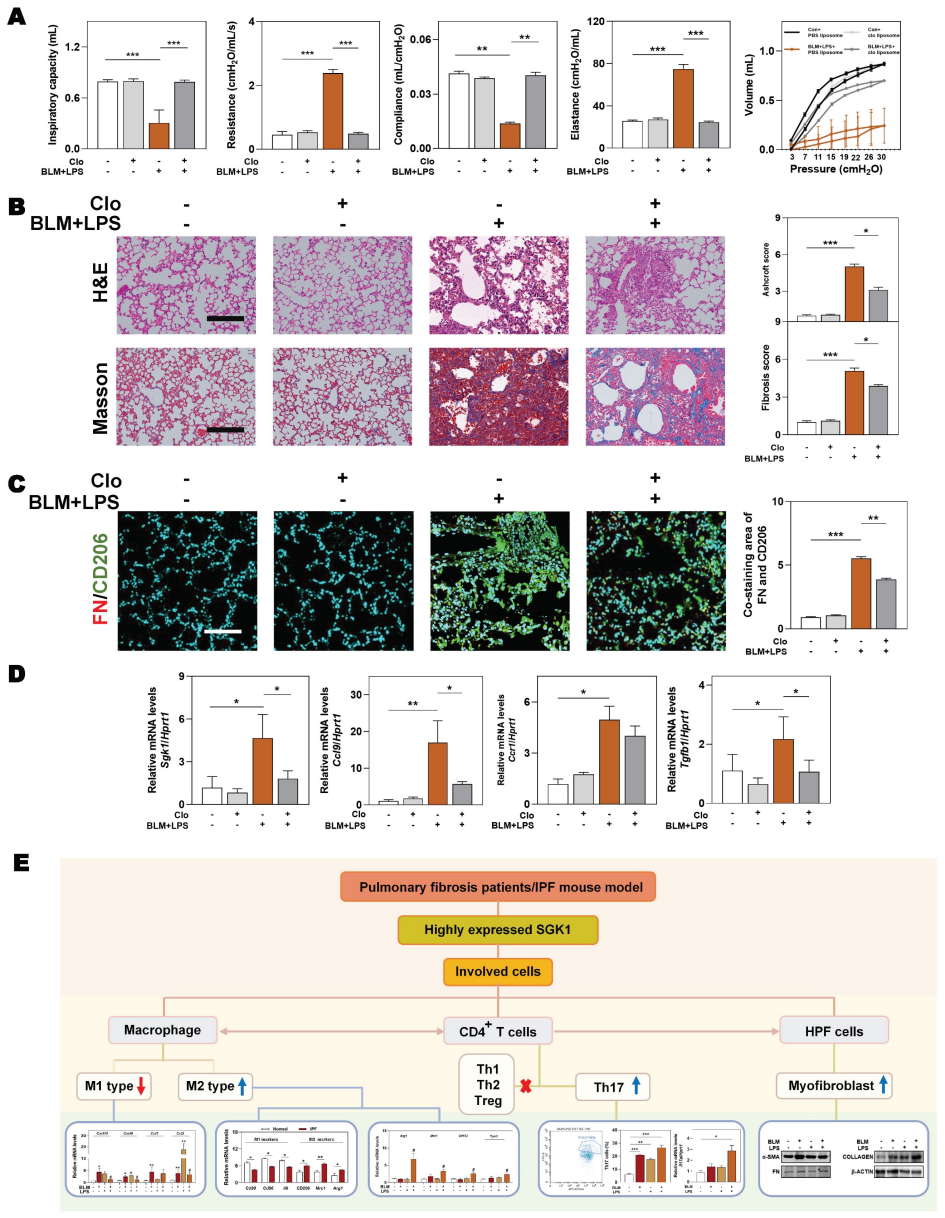
** Macrophages depletion partly ameliorates BLM plus LPS-induced experimental lung fibrosis.** Mice were administrated with BLM + LPS with or without clodronate liposomes by intratracheal instillation as described in **Method** part.** (A)** Lung inspiratory capacity, resistance, compliance, elastance and total lung pressure-volume curves.** (B)** Representative images of H&E and Masson's trichrome staining. Scale bar = 100 µm. **(C)** Immunofluorescent staining of FN and CD206 in mouse lungs. Scale bar = 200 µm. **(D)** The mRNA levels of *Sgk1, Ccl9*, *Ccr1* and *Tgfb1* and normalized with *Hprt1*.** (E)** The mind map depicting the relationship among SGK1, macrophage, Th17 cells and HPF cells. Statistical significance: **P < 0.01, ***P < 0.001, compared with relative groups (n = 6).

We further conducted a mouse model of macrophage depletion mainly in the lung by intratracheal instillation with clodronate liposomes and continued to administrate with BLM and LPS (namely Clo+ BLM + LPS group, as described in **Methods**) to investigate whether the involvement of SGK1 in IPF was dependent on macrophage transformation. Functionally, the significant increased ratios of spleen and lung weight over body weight, and the right-downward shift of pressure-volume curves were relieved in the Clo + BLM + LPS group, corresponding to a decrease of lung resistance and elastance as well as an increase of lung inspiratory capacity and compliance, when compared with BLM + LPS administrated mice (**Figure 8A** and **Figure S8A**). Consistently, histological examination revealed that the lung inflammation and fibrotic injury were largely improved in the Clo+ BLM + LPS group (**Figure 8B**). Of note, the co-immunofluorescence staining of FN and M2 macrophage maker CD206 showed a blockade M2 reprograming process after clodronate liposomes intervention (**Figure 8C** and **Figure S8B**). As shown in **Figure 8D**, the BLM + LPS-induced increase of the mRNA levels of *Sgk1*, *Ccl9*, *Ccr1* and *Tgfb1* were reversed by clodronate liposomes administration to varying degrees. Finally, we used a mind map to comb the whole process and depicting that SGK1 promoted reprograming of M2 macrophages and further increased the secretion of CCL9 and TGF-β to recruit Th17 cells and activate HPF cells, aggravating inflammatory injury and fibrosis reaction in lungs (**Figure 8E**).

## 4. Discussion

IPF is a chronic and fibrosing interstitial lung disease with rapid progression and high risk of mortality, nevertheless, the limited understanding of IPF mechanisms impedes the development of novel therapies 28. In the current study, we documented following findings by combining clinical data, animal sequencing results and a variety of molecular biology experiments both *in vivo* and *in vitro*. Firstly, SGK1 was highly increased either in the lung of patients with pulmonary fibrosis and mouse models and was positively correlated with histological characteristics of this disease (**Figure 1** and **2**). Secondly, SGK1 directly activated GSK3β, enhanced the phosphorylation of TIP60 and promoted acetylation of H3K27, thus promoting the transcription of Tgf-β and accelerating the polarization process of macrophages (**Figure 3**, **4** and** 7**). Thirdly, the reprogrammed M2-type macrophage could secrete CCL9 to recruit and stimulate Th17 cells to secrete IL-17A thus aggravating immune disorder, and simultaneously stimulate Tgf-β to activate fibroblast and further differentiated into myofibroblasts aggravating IPF damage (**Figure 5** and **6**). Fourthly, macrophage depletion reversed the reprogramming process of M1 to M2 macrophages and, to a certain extent, relieved pulmonary fibrosis (**Figure 8**). Our results highlight that SGK1-mediated GSK3β-TIP60-H3K27ac is crucial for modulating function and promoting reprograming of macrophages that further accelerate the recruitment of Th17 cells and the activation of HPF, thus participating in the IPF progression.

Inflammatory response is initially a defense system that evolved in higher organisms to protect them from infection and injury 29. However, if lung inflammation is not adequately addressed, it will trigger an uncontrolled serial reaction and become a major driver that transforms the tissue repair process into a vicious cycle then promotes the progression of pulmonary fibrosis 30. Macrophages play an important role in the development of fibrosis induced by inflammation and excessive scar formation. It has been shown that M1 macrophages are closely related to the pro-inflammatory response while M2 macrophages play a key role in anti-inflammatory and self-repair fibrotic process 31. Meanwhile, the selected IPF patient samples were closely associated with inflammatory pathological phenotypes, accompanied with higher levels of SGK1 and M2 macrophages than control samples (**Figure 1**). At the stage of lung injury induced by BLM, macrophages are polarized to the M1-type phenotype and play a pro-inflammatory role in the presence of LPS. However, accompanied by this continuously damage, macrophages are polarized to the M2-type phenotype that aim to accelerate the resolution of inflammation but can easily backfire. Furthermore, our* in vivo* results suggested that LPS intervention in the early stage of BLM administration might aggravate the injury of pulmonary fibrosis in mice (**Figure 2**). This may be due to the LPS-induced inflammatory response superimposed on the early inflammatory damage of BLM, resulting in an out-of-control inflammatory response that promoted the M1 to M2 transition. It is worthy to note that BLM alone also significantly upregulated the expression of M2 markers even without the presence of LPS (**Figure 3**). In addition, some studies have shown that LPS potently activated the migration of macrophages 32. Thus, we speculated that BLM might cause the macrophage reprogram into M2 type, start the process of over-repairing, and then induce the fibrotic reaction with the help of LPS-recruited macrophages into the injured sites (**Figure 4**). Furthermore, we also noted that M2 macrophages might also secrete excessive TGF-β to accelerate proliferation and differentiation of lung fibroblasts, of which specific mechanism will be further clarified in the future study.

GSK3, a serine/threonine protein kinase signaling molecule with two isoforms including GSK3α and GSK3β, however, the GSK3β kinase is generally considered to possess global GSK3 activity 33. In fact, the process involving kinases regulation is extremely complex, for instance, SGK1 phosphorylated GSK3 but inactivated it at the same time. The literature suggested that the activation state of GSK3 could be seen by determining either dephosphorylation of Ser9 or phosphorylation at Tyr216, to be specific, phosphorylation of Ser9 inactivated GSK3 and autophosphorylation at Tyr216 activates GSK3 34. However, it has been documented that a high fat diet model activated SGK1, thus promoted Tau pathology *via* the phosphorylation of tau at Ser214 and activated GSK-3β, forming SGK1-GSK3β-tau complex 35. In this current study, GSK3β was activated by SGK1 mainly since GSK3β behaves in an activated state on regulatory balance. Besides, how GSK3β affects downstream genes has also been a hot topic being explored, for instance, studies demonstrated that the activated GSK3 phosphorylates the Ser86 site of Tip60, which in turn activated Tip60 and then promoted the process of histone acetylation 36, 37. Here, we suggested that BLM activated SGK1 and subsequently promoted GSK3β, thereby activating Tip60 to start H3K27ac-dependent transcriptional regulation, which may also be because GSK3β behaves in an activated state upon reaching the equilibrium state (**Figure 7**). At the same time, the acetylation of H3K27 may promote gene transcription thus increase the expression and activation of SGK1 to form a positive feedback loop 38, which could further explain the progressive exacerbation of pulmonary fibrosis after BLM + LPS administration.

Although the mechanisms of fibrosis in IPF remain poorly understood, increasing studies have led to better definition of the molecular pathways that are pathologically activated in IPF. The researchers demonstrated that the overexpression of SGK1 had been observed in a variety of fibrosis diseases, including pulmonary fibrosis, renal fibrosis and hypertensive cardiac fibrosis 19. In addition, the inhibition of SGK1 by EMD638683 was found to alleviate high-fat diet-induced pulmonary fibrosis and activation of the integrin-inflammasome pathway 18. Meanwhile, to our hypothesis, the inhibition of SGK1 could attenuate or even reverse fibrotic injury caused by BLM + LPS. Although the mouse model of BLM-induced pulmonary fibrosis has been widely used in recent years as an animal model for evaluating the effects of experimental drug treatments, it is not possible to fully recapitulate and reflect the characteristics of IPF patients. In addition, due to the variety of methods of BLM stimulation (including tracheal infusion, intravenous and intraperitoneal injection, etc.), the degrees of lung injury induced by BLM are also different 39. Currently, BLM-induced pulmonary fibrosis experiments *in vivo* have been used to develop currently used antifibrotic drugs, including pirfenidone and nintedamide, however, they do not offer a cure and are associated with tolerability issues 40. Our results suggested that, compared with the BLM model, the superimposition of LPS intervention in the early phase of BLM induction has higher SGK1 expression in the lung and its pathological features are more similar to those of patients with IPF. More importantly, we think intratracheally administration of LPS for more times might be more suitable to induce the reduplicative inflammatory process and secondary lung damage to mimic patients' clinical manifestations, and therefore, the administration of LPS at the early stage of BLM induction may be regarded as a further optimization of the earlier model. Therefore, further optimizing the pulmonary fibrosis model from different drug delivery methods is an appropriate alternative strategy for later research on the therapeutic effect *in vivo* and development of new therapies, which is also the key point that we will continue to break in the future.

## 5. Conclusion

In conclusion, the critical role of SGK1 in the pathogenesis of IPF is well-illustrated. Here, we demonstrate that SGK1 induces macrophage reprograming by activating GSK3β-TIP60-H3K27ac axis, which further promotes Th17 recruitment and HPF differentiation and eventually IPF progression. Our findings provide a rationale for the discovery of novel therapeutic approaches by targeting SGK1 to ameliorate profibrotic macrophage activation in lung fibrosis.

## Supplementary Material

Supplementary figures and tables.

## Abbreviations

BLM: bleomycin
CCL9: chemokine (C-C motif) ligand 9
ERK1/2: extracellular signal-regulated kinase 1/2
GEO: gene expression omnibus
GSK3β: glycogen synthase kinase 3beta
H3K27ac: histone-3 lysine-27 acetylation
IL-1β: interleukin-1beta
IPF: idiopathic pulmonary fibrosis
KEGG: kyoto encyclopedia of genes and genomes
LPS: lipopolysaccharide
MPO: myeloperoxidase
SGK1: serum and glucocorticoid-induced kinase 1
TGF-β: transforming growth factor-beta
Th17: T helper 17
TIP60: tat-interacting protein 60
Tnf-α: tumor necrosis factor alpha

## References

[1] Li Y, Liu R, Wu J, Li X (2020). Self-eating: friend or foe?. The emerging role of autophagy in fibrotic diseases. Theranostics.

[2] Yanagihara T, Tsubouchi K, Zhou Q, Chong M, Otsubo K, Isshiki T (2023). Vascular-Parenchymal Crosstalk Promotes Lung Fibrosis Through BMPR2 Signaling. Am J Respir Crit Care Med.

[3] Lehmann M, Buhl L, Alsafadi HN, Klee S, Hermann S, Mutze K (2018). Differential effects of Nintedanib and Pirfenidone on lung alveolar epithelial cell function in ex vivo murine and human lung tissue cultures of pulmonary fibrosis. Respir Res.

[4] Raghu G, Selman M (2015). Nintedanib and pirfenidone. New antifibrotic treatments indicated for idiopathic pulmonary fibrosis offer hopes and raises questions. Am J Respir Crit Care Med.

[5] Jia K, Wu J, Li Y, Liu J, Liu R, Cai Y (2022). A novel pulmonary fibrosis murine model with immune-related liver injury. Animal Model Exp Med.

[6] van Geffen C, Deissler A, Quante M, Renz H, Hartl D, Kolahian S (2021). Regulatory Immune Cells in Idiopathic Pulmonary Fibrosis: Friends or Foes?. Front Immunol.

[7] Nie YJ, Wu SH, Xuan YH, Yan G (2022). Role of IL-17 family cytokines in the progression of IPF from inflammation to fibrosis. Mil Med Res.

[8] Isshiki T, Vierhout M, Naiel S, Ali P, Yazdanshenas P, Kumaran V Therapeutic strategies targeting pro-fibrotic macrophages in interstitial lung disease. Biochem Pharmacol. 2023: 115501.

[9] Braga TT, Agudelo JS, Camara NO (2015). Macrophages During the Fibrotic Process: M2 as Friend and Foe. Front Immunol.

[10] Wang H, Gao Y, Wang L, Yu Y, Zhang J, Liu C (2023). Lung specific homing of diphenyleneiodonium chloride improves pulmonary fibrosis by inhibiting macrophage M2 metabolic program. J Adv Res.

[11] Xia C, Razavi M, Rao X, Braunstein Z, Mao H, Toomey AC (2019). MRP14 enhances the ability of macrophage to recruit T cells and promotes obesity-induced insulin resistance. Int J Obes (Lond).

[12] Miller JE, Ahn SH, Marks RM, Monsanto SP, Fazleabas AT, Koti M (2020). IL-17A Modulates Peritoneal Macrophage Recruitment and M2 Polarization in Endometriosis. Front Immunol.

[13] Xiaobo Y, Qiang L, Xiong Q, Zheng R, Jianhua Z, Zhifeng L (2016). Serum and glucocorticoid kinase 1 promoted the growth and migration of non-small cell lung cancer cells. Gene.

[14] Lee SG, Kim D, Lee JJ, Lee HJ, Moon RK, Lee YJ (2022). Dapagliflozin attenuates diabetes-induced diastolic dysfunction and cardiac fibrosis by regulating SGK1 signaling. BMC Med.

[15] Cicenas J, Meskinyte-Kausiliene E, Jukna V, Rimkus A, Simkus J, Soderholm D (2022). SGK1 in Cancer: Biomarker and Drug Target. Cancers (Basel).

[16] Xi X, Zhang J, Wang J, Chen Y, Zhang W, Zhang X (2019). SGK1 Mediates Hypoxic Pulmonary Hypertension through Promoting Macrophage Infiltration and Activation. Anal Cell Pathol (Amst).

[17] Xi X, Liu S, Shi H, Yang M, Qi Y, Wang J (2014). Serum-glucocorticoid regulated kinase 1 regulates macrophage recruitment and activation contributing to monocrotaline-induced pulmonary arterial hypertension. Cardiovasc Toxicol.

[18] Gu X, Meng H, Peng C, Lin S, Li B, Zhao L (2023). Inflammasome activation and metabolic remodelling in p16-positive aging cells aggravates high-fat diet-induced lung fibrosis by inhibiting NEDD4L-mediated K48-polyubiquitin-dependent degradation of SGK1. Clin Transl Med.

[19] Lu RQ, Zhang YY, Zhao HQ, Guo RQ, Jiang ZX, Guo R (2022). SGK1, a Critical Regulator of Immune Modulation and Fibrosis and a Potential Therapeutic Target in Chronic Graft-Versus-Host Disease. Front Immunol.

[20] Di Cristofano A (2017). SGK1: The Dark Side of PI3K Signaling. Curr Top Dev Biol.

[21] Han X, Sun Z (2020). Epigenetic Regulation of KL (Klotho) via H3K27me3 (Histone 3 Lysine [K] 27 Trimethylation) in Renal Tubule Cells. Hypertension.

[22] Yoo G, Kim T, Chung C, Hwang DS, Lim DS (2017). The novel YAP target gene, SGK1, upregulates TAZ activity by blocking GSK3beta-mediated TAZ destabilization. Biochem Biophys Res Commun.

[23] Raghu G, Collard HR, Egan JJ, Martinez FJ, Behr J, Brown KK (2011). An official ATS/ERS/JRS/ALAT statement: idiopathic pulmonary fibrosis: evidence-based guidelines for diagnosis and management. Am J Respir Crit Care Med.

[24] Mecozzi L, Mambrini M, Ruscitti F, Ferrini E, Ciccimarra R, Ravanetti F (2020). In-vivo lung fibrosis staging in a bleomycin-mouse model: a new micro-CT guided densitometric approach. Sci Rep.

[25] Zhang F, Ayaub EA, Wang B, Puchulu-Campanella E, Li YH, Hettiarachchi SU (2020). Reprogramming of profibrotic macrophages for treatment of bleomycin-induced pulmonary fibrosis. EMBO Mol Med.

[26] Lang F, Rajaxavier J, Singh Y, Brucker SY, Salker MS (2020). The Enigmatic Role of Serum & Glucocorticoid Inducible Kinase 1 in the Endometrium. Front Cell Dev Biol.

[27] Gan W, Ren J, Li T, Lv S, Li C, Liu Z (2018). The SGK1 inhibitor EMD638683, prevents Angiotensin II-induced cardiac inflammation and fibrosis by blocking NLRP3 inflammasome activation. Biochim Biophys Acta Mol Basis Dis.

[28] Sgalla G, Biffi A, Richeldi L (2016). Idiopathic pulmonary fibrosis: Diagnosis, epidemiology and natural history. Respirology.

[29] Singh N, Baby D, Rajguru JP, Patil PB, Thakkannavar SS, Pujari VB (2019). Inflammation and cancer. Ann Afr Med.

[30] Savin IA, Zenkova MA, Sen'kova AV (2022). Pulmonary Fibrosis as a Result of Acute Lung Inflammation: Molecular Mechanisms, Relevant In Vivo Models, Prognostic and Therapeutic Approaches. Int J Mol Sci.

[31] Locati M, Curtale G, Mantovani A (2020). Diversity, Mechanisms, and Significance of Macrophage Plasticity. Annu Rev Pathol.

[32] Tu Y, Zhang L, Tong L, Wang Y, Zhang S, Wang R (2018). EFhd2/swiprosin-1 regulates LPS-induced macrophage recruitment via enhancing actin polymerization and cell migration. Int Immunopharmacol.

[33] Hoffmeister L, Diekmann M, Brand K, Huber R (2020). GSK3: A Kinase Balancing Promotion and Resolution of Inflammation. Cells.

[34] Krishnankutty A, Kimura T, Saito T, Aoyagi K, Asada A, Takahashi SI (2017). In vivo regulation of glycogen synthase kinase 3beta activity in neurons and brains. Sci Rep.

[35] Elahi M, Motoi Y, Shimonaka S, Ishida Y, Hioki H, Takanashi M (2021). High-fat diet-induced activation of SGK1 promotes Alzheimer's disease-associated tau pathology. Hum Mol Genet.

[36] Charvet C, Wissler M, Brauns-Schubert P, Wang SJ, Tang Y, Sigloch FC (2011). Phosphorylation of Tip60 by GSK-3 determines the induction of PUMA and apoptosis by p53. Mol Cell.

[37] Sun Y, Jiang X, Chen S, Fernandes N, Price BD (2005). A role for the Tip60 histone acetyltransferase in the acetylation and activation of ATM. Proc Natl Acad Sci U S A.

[38] Cheng D, Dong Z, Lin P, Shen G, Xia Q (2022). Transcriptional Activation of Ecdysone-Responsive Genes Requires H3K27 Acetylation at Enhancers. Int J Mol Sci.

[39] Della Latta V, Cecchettini A, Del Ry S, Morales MA (2015). Bleomycin in the setting of lung fibrosis induction: From biological mechanisms to counteractions. Pharmacol Res.

[40] Spagnolo P, Kropski JA, Jones MG, Lee JS, Rossi G, Karampitsakos T (2021). Idiopathic pulmonary fibrosis: Disease mechanisms and drug development. Pharmacol Ther.

