# Supplementary material for: SGK1 aggravates idiopathic pulmonary fibrosis by triggering H3k27ac-mediated macrophage reprogramming and disturbing immune homeostasis
Source: Int J Biol Sci. 2024 Jan 12;20(3):968–86. doi: 10.7150/ijbs.90808 (PMC10797695; doi:10.7150/ijbs.90808)
Supplement: Supplementary file 1 — Supplementary figures and tables. [file ijbsv20p0968s1.pdf]

SGK1 aggravates idiopathic pulmonary fibrosis by triggering H3k27ac-mediated macrophage reprogramming and disturbing immune homeostasis

Jianzhi Wu[1, #], Liping Gong [3, #], Yijie Li[1], Tiegang Liu[2, 5], Rong Sun[3], Kexin Jia[1], Runping Liu[4], Fei Dong[2, 5], Xiaohong Gu[2, 5, *], Xiaojiaoyang Li[1, *]

[1] School of Life Sciences, Beijing University of Chinese Medicine, Beijing, 100029, China

[2] Institute of Chinese Epidemic Disease, Beijing University of Chinese Medicine, Beijing 100029, China

[3] The Second Hospital of Shandong University, Shan Dong University, 247 Bei Yuan Da Jie, Jinan, 250033, China

[4] School of Chinese Materia Medica, Beijing University of Chinese Medicine, 11 Bei San Huan Dong Lu, Beijing, 100029, China

[5] School of Traditional Chinese Medicine, Beijing University of Chinese Medicine, Beijing, 100029, China

#These authors contributed equally to this work.

***Corresponding authors:** Prof. Xiaojiaoyang Li and Prof. Xiaohong Gu

Address correspondence to:

Xiaojiaoyang Li, Ph.D.

School of Life Sciences

Beijing University of Chinese Medicine, Beijing, China

Email: xiaojiaoyang.li@bucm.edu.cn

OR

Xiaohong Gu, MD, Ph.D.

Institute of Chinese Epidemic Disease

Beijing University of Chinese Medicine

Email: guxh1003@126.com

Supplemental figure legends

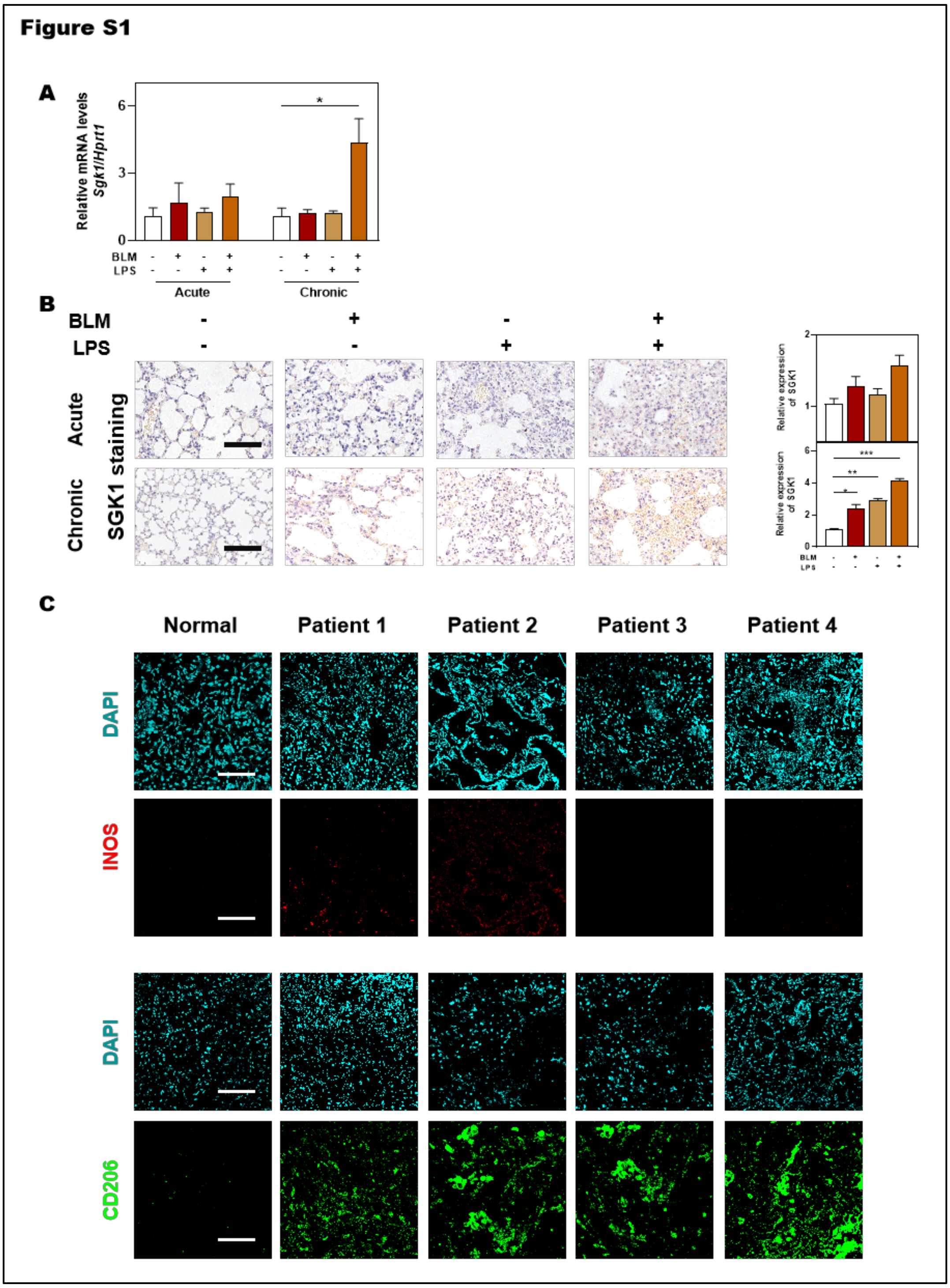

Figure S1. Expressions of SGK1 in BLM- and LPS- induced acute and chronic IPF mice models. (**A**) The mRNA levels of *Sgk1* in acute and chronic IPF mice. (**B**) Representative images of immunohistochemistry staining of SGK1 in acute and chronic IPF mice lungs (Scale bar = 100 μm). (**C**) Representative images of

immunofluorescent staining of iNOS and CD206 in normal and IPF patient lungs. Scale bar = 200 μm. Statistical significance: **P*<0.05, ***P*<0.01, ****P*<0.001, compared with relative groups (n=6).

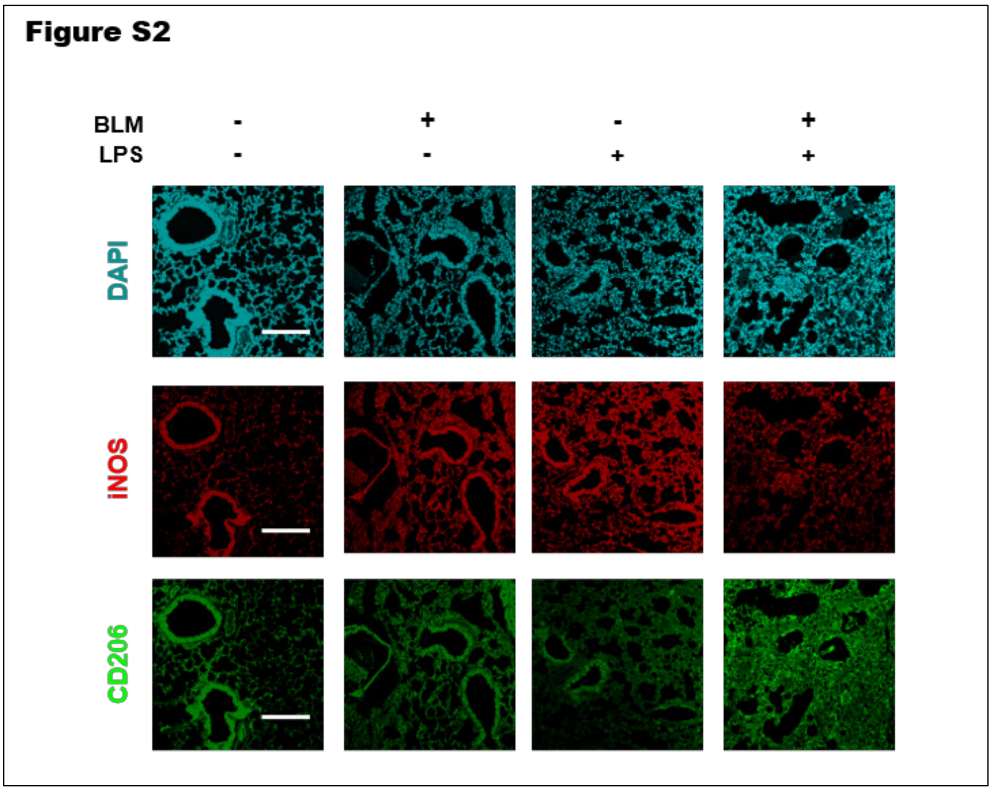

Figure S2. BLM + LPS promotes the reprograming of macrophages from M1 to M2. Representative images of immunofluorescent staining of iNOS and CD206 in lungs. Scale bar = 200 μm.

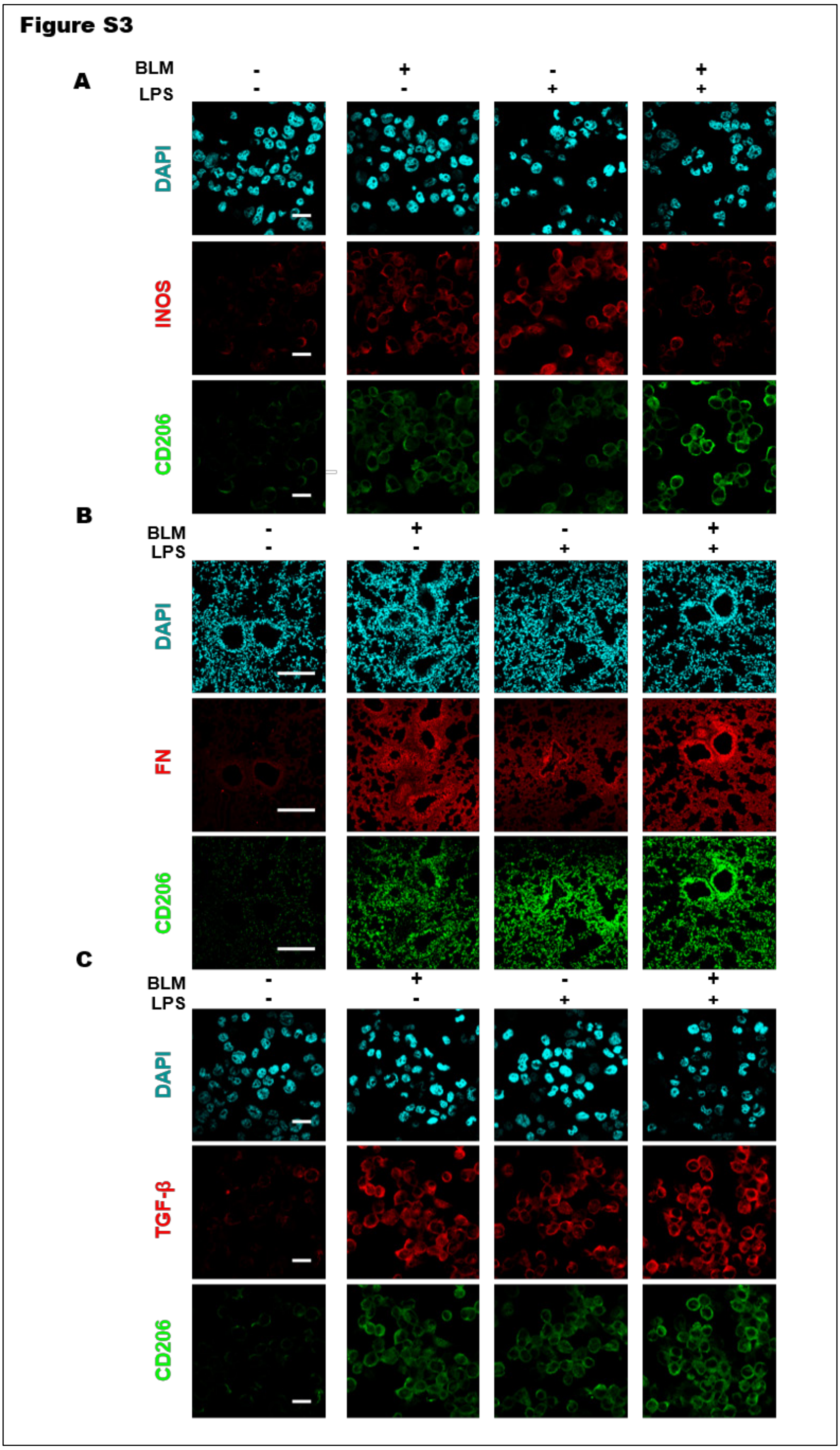

Figure S3. BLM plus LPS increases the number of M2 macrophages, aggravates

fibrotic injury along with increases TGF-β1 secretion. Representative images of immunofluorescent co-staining of iNOS and CD206 **(A)** in THP-1 cells (Scale bar = 50 μm), and co-staining of FN and CD206 **(B)** in lung tissues (Scale bar = 200 μm). **(C)** Representative images of immunofluorescent result of TGF-β and CD206 in THP-1 cells treated with BLM and/or LPS. Scale bar = 50 μm.

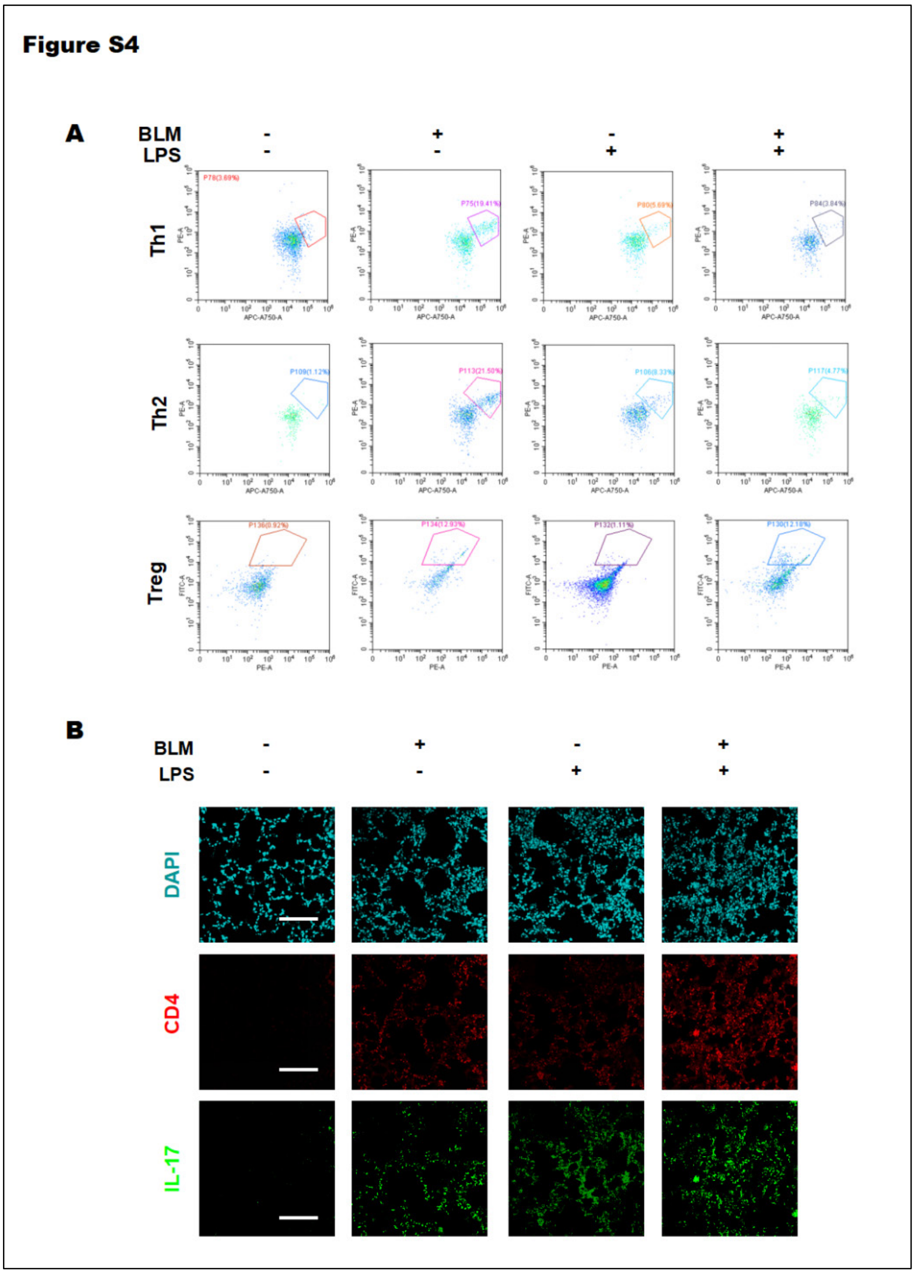

Figure S4. BLM plus LPS increases the number of Th17 cells. (A) Flow cytometry analyzes number of Th1, Th2 and Treg cells in the lung. **(B)** Representative images of

immunofluorescent result of CD4 and IL-17 in lungs. Scale bar = 200 μm.

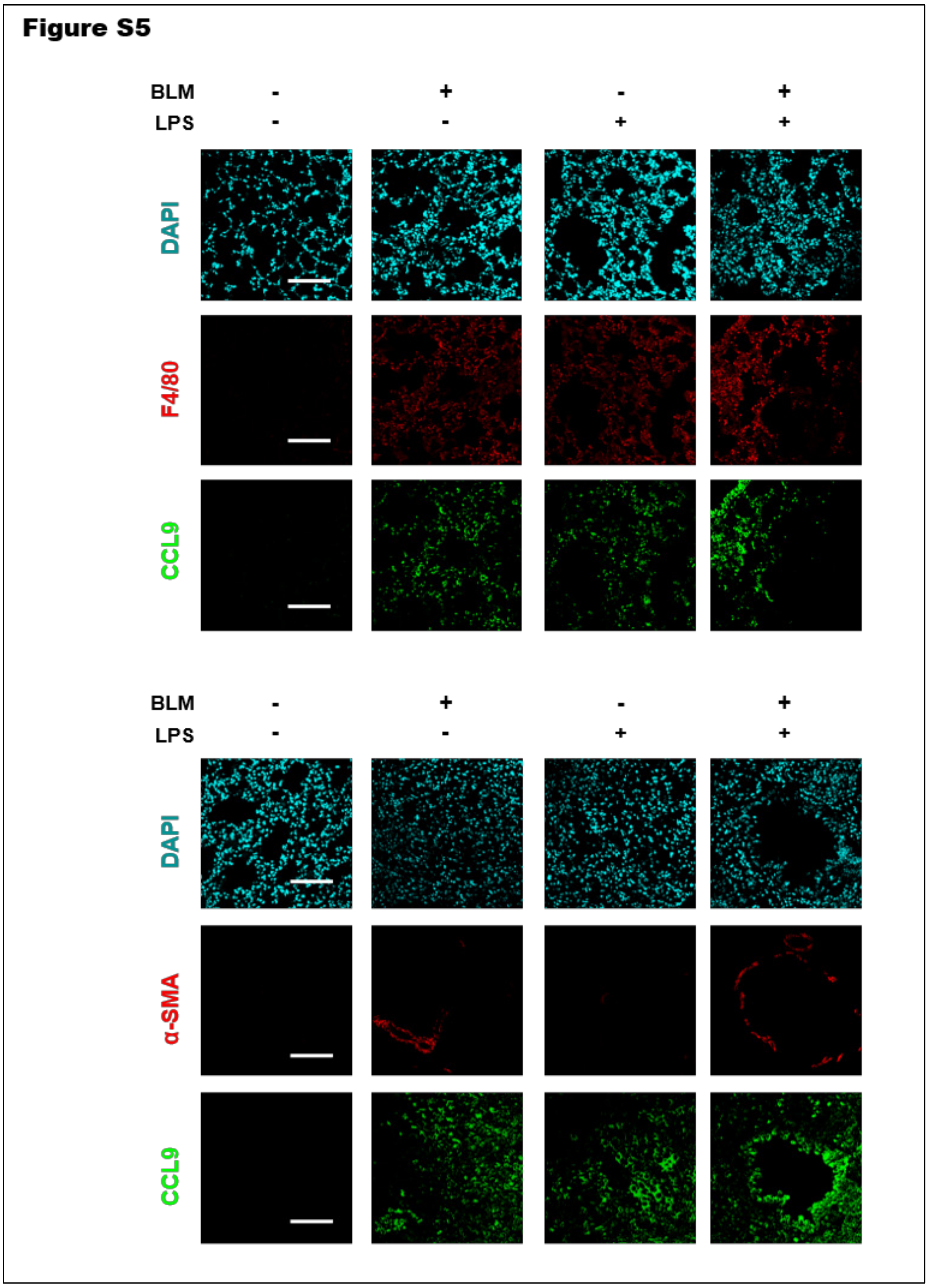

Figure S5. Macrophage rather than the myofibroblasts were the main producers of CCL9 in fibrotic lung tissues. Representative images of co-immunofluorescent staining of CCL9 with F4/80 and α-SMA. Scale bar = 200 μm.

Figure S6

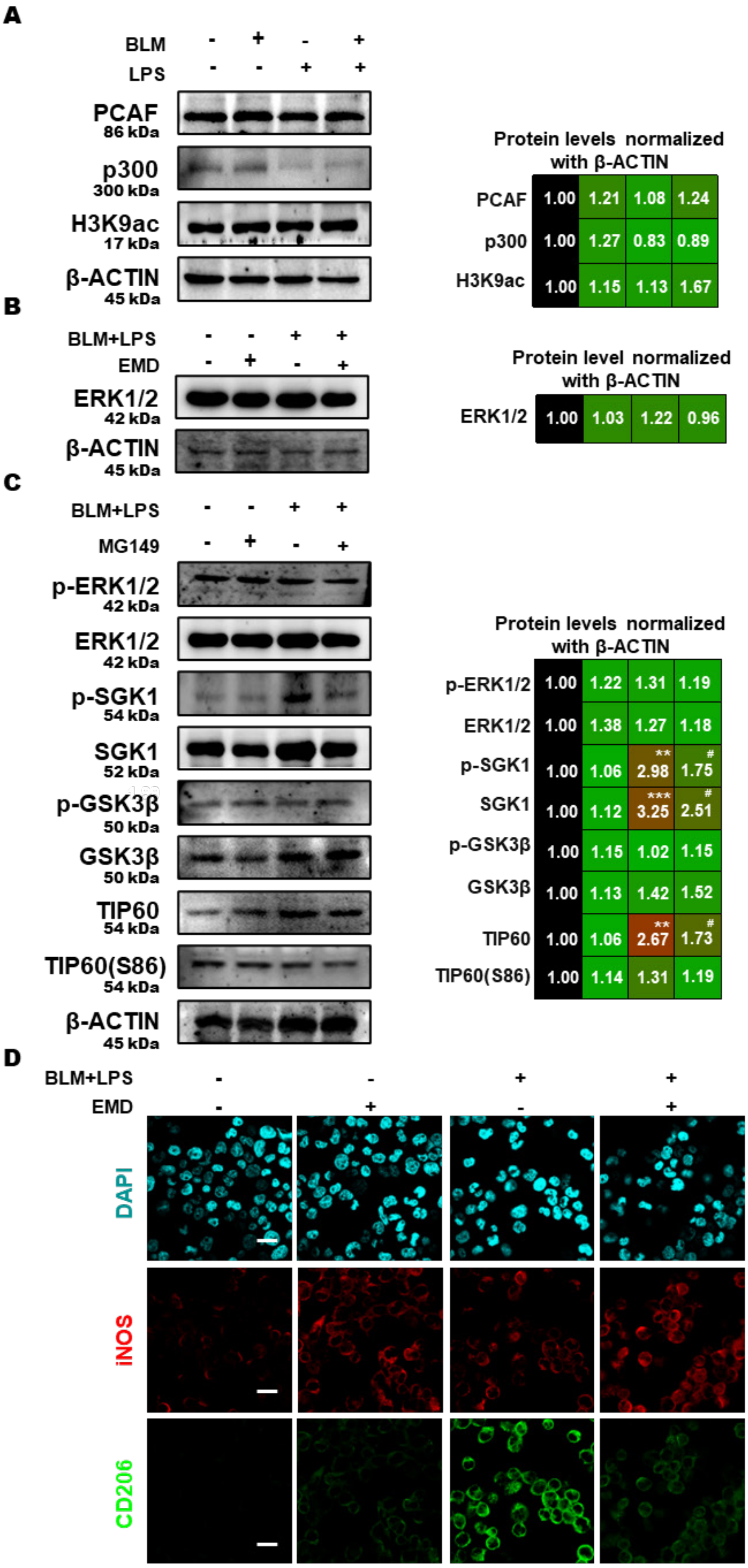
A
BLM
LPS
PCAF 86 kDa
p300 300 kDa
H3K9ac 17 kDa
β-ACTIN 45 kDa
Protein levels normalized with β-ACTIN
PCAF 1.00 1.21 1.08 1.24
p300 1.00 1.27 0.83 0.89
H3K9ac 1.00 1.15 1.13 1.67
B
BLM+LPS
EMD
ERK1/2 42 kDa
β-ACTIN 45 kDa
Protein level normalized with β-ACTIN
ERK1/2 1.00 1.03 1.22 0.96
C
BLM+LPS
MG149
p-ERK1/2 42 kDa
ERK1/2 42 kDa
p-SGK1 54 kDa
SGK1 52 kDa
p-GSK3β 50 kDa
GSK3β 50 kDa
TIP60 54 kDa
TIP60(S86) 54 kDa
β-ACTIN 45 kDa
Protein levels normalized with β-ACTIN
p-ERK1/2 1.00 1.22 1.31 1.19
ERK1/2 1.00 1.38 1.27 1.18
p-SGK1 1.00 1.06 ** 2.98 # 1.75
SGK1 1.00 1.12 *** 3.25 # 2.51
p-GSK3β 1.00 1.15 1.02 1.15
GSK3β 1.00 1.13 1.42 1.52
TIP60 1.00 1.06 ** 2.67 # 1.73
TIP60(S86) 1.00 1.14 1.31 1.19
D
BLM+LPS
EMD
DAPI
iNOS
CD206

Figure S6. Macrophage reprogramming is regulated by SGK1-mediated GSK3β-TIP60 pathway. Representative immunoblots against ERK1/2 **(A)**, PCAF, p300, K3K9ac **(B)**, p-ERK1/2, ERK1/2, p-SGK1, SGK1, p-GSK3β, GSK3β, TIP60, TIP60 (S86) and β-ACTIN **(C)** in THP-1 cells. **(D)** Representative images of immunofluorescent result of iNOS and CD206 in THP-1 cells. Scale bar = 50 μm. Statistical significance: $^{**}P < 0.01$, $^{***}P < 0.001$, compared with control group; $^{\#}P < 0.05$, compared with BLM + LPS group (n = 3).

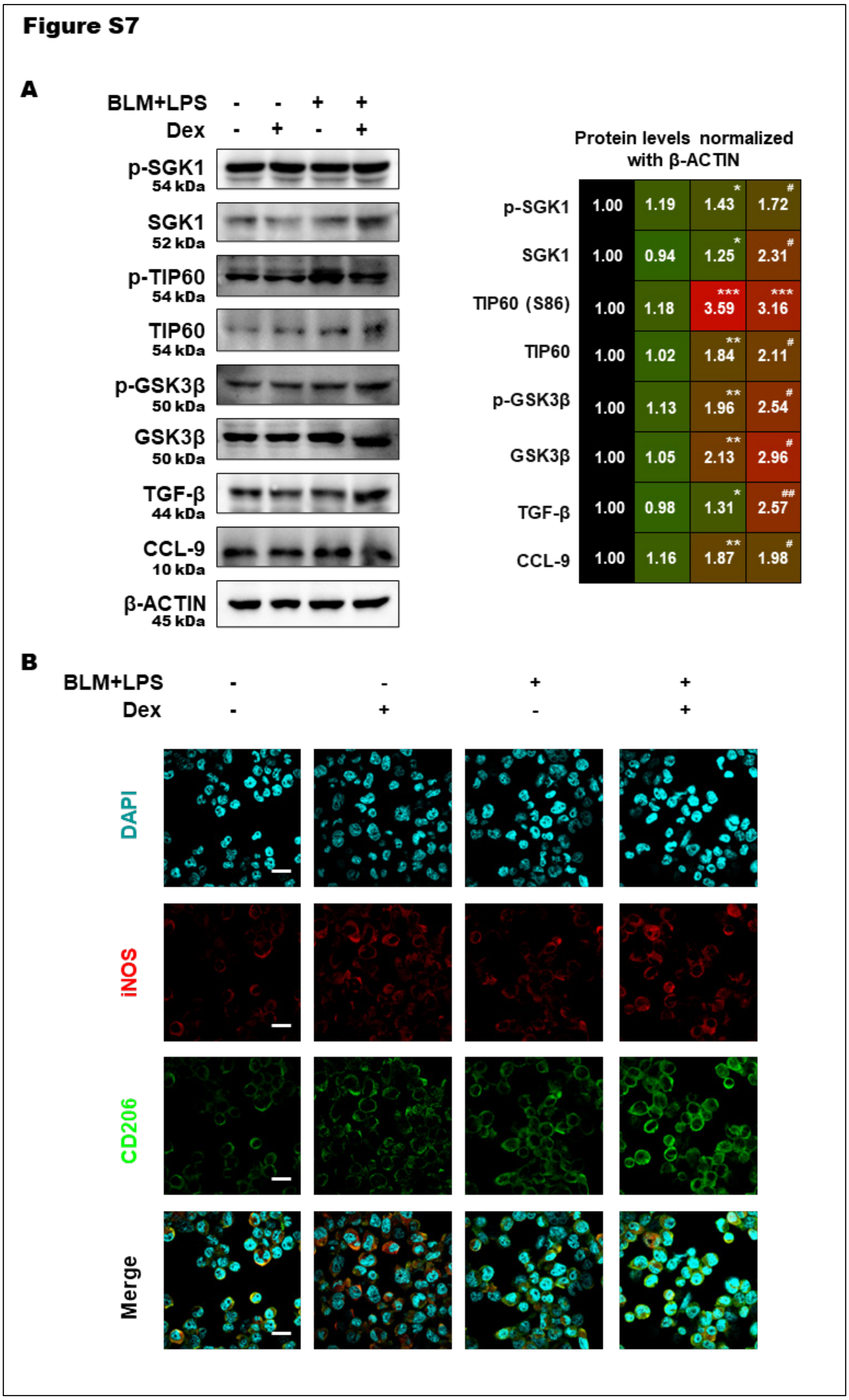

Figure S7. Macrophage reprogramming is promoted by activating SGK1. (A)

Representative immunoblots against p-SGK1, SGK1, p-GSK3β, GSK3β, TIP60, TIP60

(S86), TGF-β, CCL-9 and β-ACTIN in THP-1 cells. **(B)** Representative images of immunofluorescent result of iNOS and CD206 in THP-1 cells. Scale bar = 50 μm. Statistical significance: **P* < 0.05, ***P* < 0.01, ****P* < 0.001, compared with control group; $^{\#}P < 0.05$, $^{\#\#}P < 0.01$, compared with BLM + LPS group (n = 3).

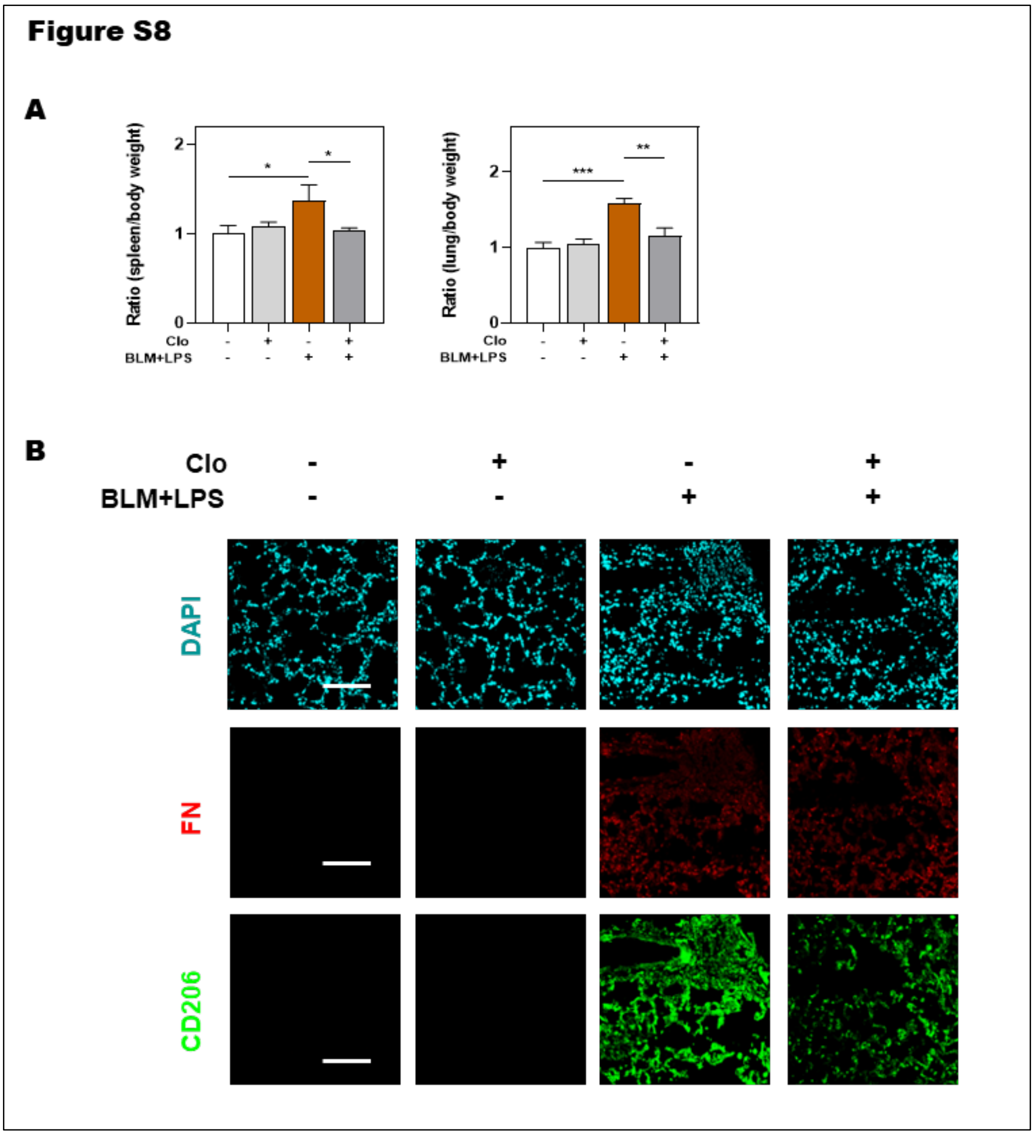

Figure S8. Lung fibrotic injury and M2 reprograming process are largely improved after Clo intervention in BLM + LPS induced IPF mice. (A) Ratio of spleen and lung to body weight. **(B)** Immunofluorescent staining of FN and CD206 in

mouse lungs. Scale bar = 200 μm.

Table S1 The baseline characteristics of the IPF patients.

**Characteristics**	**Values (*n* = 27)**
Age（y）	56.44±8.71
Sex (male/female)	14/13
BMI (kg/m^2)	25.67±3.54
Smoking status	
Current smoker	3
Exsmoker	1
Nonsmoker	23
Exposure history (house dust,birds, cotton, chemical materials)	
None	27
Yes	0
System disease (dermatomyositis, rheumatoid arthritis, sarcoidosis, lupus & scleroderma)	
None	27
Yes	0
Pulmonary function tests	
FVC	3.15±0.89
% FVC	95.04±18.07
FEV_1	2.51±0.81
% FEV_1	91.94±20.22
FEV_1/FVC (%)	78.98±10.00
% DL_{CO}	88.74±16.83
% DL_{CO}/V_A	102.11±18.98
Echocardiography	
% Ejection fraction	63.63±3.28

Notes: Data presented as *n* or mean ± standard deviation, unless otherwise indicated.

Abbreviations: DL_{CO} = diffusing capacity of the lung for carbon dioxide, FEV_1 = forced expiratory volume in 1 s, FVC = forced vital capacity, V_A = alveolar volume.

Table S2 The antibodies used in our study.

Antibody	Catalog Number	Vendor
FIBRONECTIN	15613-1-AP	Proteintech Group (Rosemont, USA)
E-Cadherin	20874-1-AP	Proteintech Group
SGK1	28454-1-AP	Proteintech Group
iNOS	18985-1-AP	Proteintech Group
TGF beta1	21898-1-AP	Proteintech Group
CD206	60143-1-Ig	Proteintech Group
Histone-H3	17168-1-AP	Proteintech Group
F4/80	28463-1-AP	Proteintech Group
Phospho-GSK3β	29125-1-AP	Proteintech Group
GSK-3β	sc-377213	Santa Cruz Biotechnology (Texas, USA)
PCAF	sc-13124	Santa Cruz Biotechnology
TIP60	sc-32244	Santa Cruz Biotechnology
p300	sc-32244	Santa Cruz Biotechnology
MIP-1γ	sc-74228	Santa Cruz Biotechnology
IL-17	sc-374218	Santa Cruz Biotechnology
APC anti-mouse cluster of differentiation 3 antibody	100236	BIOLEGEND (Beijing, China)
APC/Cyanine7 anti-mouse CD4 antibody	100413	BIOLEGEND
FITC anti-mouse IL-17A antibody	506907	BIOLEGEND
alpha-smooth muscle actin	19245S	Cell Signaling Technology (Danvers, USA)
Phospho-SGK1	5599S	Cell Signaling Technology
Acetyl-Histone H3	4353S	Cell Signaling Technology
β-ACTIN	4970S	Cell Signaling Technology
Tip60	ab73207	Abcam (Melbourne, VIC, Australia)
Collagen 1	ab34710	Abcam
Goat anti-mouse IgG (H+L) Highly Cross-Adsorbed secondary antibody (Alexa Fluor Plus 488)	UI287767	Thermo Fisher Scientific
Goat anti-rabbit IgG (H+L), F(ab')2 Fragment (Alexa Fluor 594 Conjugate)	8889S	Cell Signaling Technology
